# Diverse susceptibilities and responses of human and rodent cells to orthohantavirus infection reveal different levels of cellular restriction

**DOI:** 10.1371/journal.pntd.0010844

**Published:** 2022-10-12

**Authors:** Giulia Gallo, Petr Kotlik, Philippe Roingeard, Marc Monot, Guillaume Chevreux, Rainer G. Ulrich, Noël Tordo, Myriam Ermonval

**Affiliations:** 1 Institut Pasteur, Université Paris Cité, Département de Virologie, Unité des Stratégies Antivirales, Paris, France; 2 Sorbonne Université, Ecole Doctorale Complexité du Vivant, Paris, France; 3 Laboratory of Molecular Ecology, Institute of Animal Physiology and Genetics, Czech Academy of Sciences, Liběchov, Czech Republic; 4 INSERM U1259 et plateforme IBISA de Microscopie Electronique, Université et CHRU de Tours, Tours, France; 5 Institut Pasteur, Université Paris Cité, Biomics Platform, C2RT, Paris, France; 6 Université Paris Cité, CNRS, Institut Jacques Monod, Paris, France; 7 Institute of Novel and Emerging Infectious Diseases, Friedrich-Loeffler-Institut, Partner site Hamburg-Lübeck-Borstel-Riems, German Centre for Infection Research (DZIF), Greifswald-Insel Riems, Germany; 8 Institut Pasteur de Guinée, Conakry, Guinée; Karolinska Institutet, SWEDEN

## Abstract

Orthohantaviruses are rodent-borne emerging viruses that may cause severe diseases in humans but no apparent pathology in their small mammal reservoirs. However, the mechanisms leading to tolerance or pathogenicity in humans and persistence in rodent reservoirs are poorly understood, as is the manner in which they spread within and between organisms. Here, we used a range of cellular and molecular approaches to investigate the interactions of three different orthohantaviruses–Puumala virus (PUUV), responsible for a mild to moderate form of hemorrhagic fever with renal syndrome in humans, Tula virus (TULV) with low pathogenicity, and non-pathogenic Prospect Hill virus (PHV)–with human and rodent host cell lines. Besides the fact that cell susceptibility to virus infection was shown to depend on the cell type and virus strain, the three orthohantaviruses were able to infect Vero E6 and HuH7 human cells, but only the former secreted infectious particles. In cells derived from PUUV reservoir, the bank vole (*Myodes glareolus)*, PUUV achieved a complete viral cycle, while TULV did not enter the cells and PHV infected them but did not produce infectious particles, reflecting differences in host specificity. A search for mature virions by electron microscopy (EM) revealed that TULV assembly occurred in part at the plasma membrane, whereas PHV particles were trapped in autophagic vacuoles in cells of the heterologous rodent host. We described differential interactions of orthohantaviruses with cellular factors, as supported by the cellular distribution of viral nucleocapsid protein with cell compartments, and proteomics identification of cellular partners. Our results also showed that interferon (IFN) dependent gene expression was regulated in a cell and virus species dependent manner. Overall, our study highlighted the complexity of the host-virus relationship and demonstrated that orthohantaviruses are restricted at different levels of the viral cycle. In addition, the study opens new avenues to further investigate how these viruses differ in their interactions with cells to evade innate immunity and how it depends on tissue type and host species.

## Author summary

Orthohantaviruses are zoonotic RNA viruses found all over the world in association with small mammal reservoirs. When occasionally transmitted to humans by aerosol they can cause two main types of diseases: hemorrhagic fever with renal syndrome (HFRS) mainly in Europe and Asia and hantavirus cardiopulmonary syndrome (HCPS) in North and South America with a case fatality rate of up to 15% and 40% respectively. An increasing number of outbreaks are recorded in endemic areas and, with disturbance of rodent habitats, climate change and the risk of virus genome reassortment, the emergence of new orthohantaviruses is of concern. With no treatment, no vaccines and few molecular tools, little is known about the pathophysiology of these viruses. Our comparative study of the viral cycle and interaction of different pathogenic and low or non-pathogenic orthohantaviruses in cells derived from human or rodent hosts reveals differences in entry, RNA replication or release of infectious particles, concurrently to regulation of host genes. This study illustrates how the development of a rodent host cell model together with the availability of an annotated bank vole genome may contribute to a better understanding of mechanisms of the interactions between orthohantaviruses and their hosts.

## Introduction

Orthohantaviruses are emerging viruses hosted by small mammals, such as rodents and shrews, with which they have co-evolved for a long time [1]. Because of the global distribution of their animal reservoirs, orthohantaviruses are found on all inhabited continents of our planet. To date, only orthohantaviruses specifically infecting rodents have been identified as causing human diseases upon viral transmission through inhalation when humans come into contact with aerosolized excreta of infected animal reservoirs. Recently, orthohantaviruses have been involved in recurrent human epidemics [2]. According to the orthohantavirus species and affected organs in humans, two different diseases have been described: the hantavirus cardiopulmonary syndrome (HCPS) exclusively in the Americas, and the so-called hemorrhagic fever with renal syndrome (HFRS) mainly in Eurasia. First, viruses enter the respiratory tract and then propagate to different organs, in particular lung, kidney and liver, whose physiology is altered in infected patients. Even though the precise mechanism of propagation is not completely understood, it is known that vascular leakage and thrombocytopenia contribute to both HCPS and HFRS, probably due to bursts of cytokines produced by infected endothelial cells and by immune cells recruited at the site of infection [3–5].

In contrast to the human host, which can be occasionally infected, animal reservoirs are persistently infected by orthohantaviruses throughout their life span without any obvious sign of pathology [6]. In these natural hosts, viruses are transmitted through inhalation of virus contaminated aerosols and wound contact with infected saliva during aggressive behavior associated with mating. Viral RNA can be found in different organs of rodents and has been detected in lungs, kidneys, liver, brain and gallbladder, but importantly is excreted in urine and feces of infected animals [7,8].

In human and rodent hosts, orthohantaviruses primarily infect epithelial and endothelial cells and also macrophages and dendritic cells, which could promote the propagation of viruses in the organism [9]. It is thought that the first step for cellular susceptibility to viral infection could rely on interaction with different integrins, used as primary receptors, depending on the virus origin and its pathogenicity [10,11]. However, a recent study has shown that protocadherin-1 is crucial for infection of endothelial cells by the pathogenic New World orthohantaviruses, Andes virus (ANDV) and Sin Nombre virus (SNV), and suggests that integrins probably only play minor roles in cellular infection [12]. In support, bank vole-derived immortalized cells express low levels of integrin-β3 [13] and virions can be internalized by clathrin-dependent [14] and independent [15] mechanisms, as well as by macropinocytosis [16]. The replication cycle of the three RNA segments of negative polarity, S, M and L, of orthohantaviruses then entirely takes place in the cytoplasm. These three genome segments encode the nucleocapsid (N), the RNA polymerase and the two envelope glycoproteins, Gn and Gc, generated by cleavage of a glycoprotein precursor, GPC. Maturation and assembly of Gn/Gc complexes take place in the secretory pathway [17]. Then, the cytoplasmic ribonucleoproteins (RNP), consisting of viral RNA segments wrapped into N protein oligomers, interact with the Gn glycoprotein cytosolic tails (GnCT) at the Golgi site of viral particle assembly and budding [18,19]. However, in contrast to Old World orthohantaviruses, some orthohantaviruses from the Americas could bud directly at the plasma membrane [20].

As suggested by their low number, structural hantaviral proteins may harbor multiple functions to successfully accomplish a viral cycle. The viral N protein not only protects the viral RNA and acts in viral replication and transcription, but also interacts with multiple cellular factors [21], is associated to the endoplasmic reticulum-Golgi intermediate compartment, ERGIC [22] and regulates cellular processes such as mRNA post-transcription decay [23], apoptosis [24–26] and interacts with the cytoskeleton network [27]. In the case of envelope glycoproteins, in addition to their roles in entry, trafficking and maturation of viral particles, they could also contribute to innate immunity through interaction of their long cytosolic tails with cellular factors. The GnCT has indeed been shown to activate cellular kinases implicated in the regulation of endothelial and immune functions [28,29]. An antagonistic role to interferon antiviral activity has also been demonstrated, which could be linked to the pathogenicity of orthohantaviruses [30,31].

Due to the lack of molecular tools, including reverse genetics systems and, annotated genome, transcriptome and proteome libraries for each animal reservoir, few studies have been conducted on the viral life cycle during orthohantavirus infection. Therefore, only fragmentary information is available [32], especially on the interactions of orthohantaviruses with the immune system of their rodent reservoirs [9,33]. Furthermore, due to the fact that orthohantaviruses cause persistent asymptomatic infection in rodent hosts, relevant animal models of pathogenicity are missing, in particular for Old World orthohantaviruses [34]. Information on the role of endothelial and immune cells in pathogenesis comes mainly from studies on the activated factors found in infected cells and in patients with HFRS or HCPS [35].

In order to bring information on mechanisms of pathogenicity and persistence of orthohantaviruses in their different hosts, we performed comparative studies of the interactions carried out in human and rodent cell lines by three orthohantaviruses: human pathogenic Puumala virus (PUUV), low or non-pathogenic Tula virus (TULV) and Prospect Hill virus (PHV). PUUV is one of the most important European orthohantavirus, which is responsible for nephropathia epidemica, a mild to moderate form of HFRS, and is hosted by the bank vole (*M*. *glareolus* syn. *Clethrionomys glareolus)*. PHV is considered as a viral model of apathogenic orthohantaviruses and is associated to the meadow vole (*Microtus pennsylvanicus*), from North America. TULV is found in Europe and is hosted by the common vole (*Microtus arvalis*), although it was also molecularly detected in other related vole species [36]. It is considered to be of low pathogenicity due to the rare report of human cases [37,38].

We hypothesized that characterizing differences in the capacity of orthohantaviruses to infect cells, interact with cellular factors, regulate cellular functions and propagate, will provide insight into different outcomes of orthohantavirus infection. Therefore, our objectives were to compare the interactions of pathogenic (PUUV) and low and non-pathogenic (TULV and PHV) orthohantaviruses with cells derived from the human host, and the bank vole, with a particular effort in studying rodent cells-virus relationships, to date poorly described. Moreover, due to the apparent conflicting results present in the literature (usually focused on one virus or one cell line), we thought it would be important to address these questions by designing a comprehensive study (multiple cell lines, multiple viruses) and by including different omics approaches, scarcely reported for orthohantaviruses in the literature. The ability of the viruses to replicate and produce infectious particles was assessed by monitoring the intracellular expression of N protein during primary and secondary infections and by quantifying the copy number of viral genomes produced by infected cells. EM was used to detect mature viral particles in infected cells and immunofluorescence staining to follow viral interaction and remodeling of cellular compartments. This prompted us to investigate cellular interactors of viral proteins by mass-spectrometric identification and search for transcription signatures in bank vole cells infected by orthohantaviruses, as compared to human cells, performing RNA-sequencing (RNA-Seq) and real time-quantitative PCR (RT-qPCR). Altogether our data highlight different levels of cellular restriction to orthohantaviral infection, which can be linked with the observed regulation of gene expression depending on the virus, the cell type and its host origin raising questions about innate immune responses of infected cells. These differential interactions are discussed in the context of the outcomes of orthohantavirus infections.

## Materials and methods

### Cells and viruses

Vero E6 cells, kidney epithelial cells from African green monkey, were grown in Dulbecco’s Modified Eagle’s Medium (DMEM). HuH7 (human hepatocarcinoma), A549 (human lung carcinoma, ATCC-CCL-185) and Caco-2 (human colorectal adenocarcinoma) cell lines were maintained in DMEM supplemented with non-essential amino acids and 1 mM sodium pyruvate. The human monocyte THP-1 cell line was maintained in suspension in RPMI 1640 Medium. Cells were treated with phorbol 12-myristate 13-acetate (PMA, Sigma-Aldrich) at 100 nM in Macrophage-SFM medium for 24 or 48 hours, in order to induce their differentiation in macrophages. Human umbilical vascular endothelial cells, HUVEC (a kind gift from Philippe Afonso, Institut Pasteur, Paris) were grown in EndoGRO-MV complete media kit (Merck). Media and additives were purchased from GIBCO (Invitrogen) except when otherwise indicated.

Bank vole cells derived from different organs of *M*. *glareolus* were immortalized by the large T antigen of Simian virus 40 (SV40), as described [39]. The different bank vole cell lines, generated within the EVAg project by Charité (Berlin, Germany) were kindly provided by Isabella Eckerle and Marcel Müller (Bonn University, Germany): MyglaSWRecB and MyglaSWTrach cells are epithelial cells from kidneys and from the tracheal apparatus respectively and MyglaAECcl2 cell clone is derived from alveolar lung epithelial cells. BVK168 is a spontaneously immortalized bank vole kidney cell line [40], kindly provided by Sandra Essbauer (Bundeswehr Institute of Microbiology, Munich, Germany). All these cells were cultured in DMEM. MH-S, a house mouse (*Mus musculus*) alveolar macrophage cell line (ATCC-CRL-2019), was grown in RPMI 1640.

Except for HUVEC, the different media used to maintain human and vole cells were supplemented with 10% of heat-inactivated fetal bovine serum (FBS), purchased from Biosera. All cell lines were kept growing at 37°C, 5% CO_2_ in humid atmosphere. Cells were mycoplasma free, as determined by Mycoalert test (Lonza)

PUUV strain Sotkamo and PHV strain 3571, as well as Vero E6 cells, were kindly provided by Andreas Rang (Charité Berlin, Germany). TULV strain Moravia was obtained from Alexander Plyusnin (Helsinki University, Finland). Virus stocks were prepared on Vero E6 cells seeded in 75 cm^2^ culture flasks, the day before infection to let cells adhere to the surface. Virus was added for 1 h on the cell layer at a multiplicity of infection (MOI) around 0.1 in 2 ml of DMEM 5% FBS. Twenty mL of DMEM/5% FBS were then added. Culture supernatants were recovered 6- (TULV) or 7- (PUUV, PHV) days post infection (dpi) and kept frozen in aliquot at -80°C.

### Antibodies

Viral N protein was detected using a commercial mouse monoclonal antibody (A1C5, antibodies-online) raised against the N protein of PUUV-CG18-20 strain, while a home-made rabbit polyclonal antibody raised against the ectodomain of PUUV-Gn was used to detect PUUV, TULV and PHV glycoproteins [30]. The following rabbit polyclonal antibodies were used to detect cellular compartments: anti-calnexin for the endoplasmic reticulum, anti-LMAN1 for the ERGIC, anti-giantin for the Golgi, and Golgin 97 for the Trans Golgi Network, all provided by Novus Bio, while anti-EEA1 from Cell Signaling and anti-Rab7 from Abcam were used to detect early and late endosomes, respectively. Anti-DDX6 (Novus Bio) was used to detect P-bodies. Cytoskeleton filaments were detected with anti-tubulin (Novus Bio) and anti-vimentin (Abcam) and actin filaments were visualized using Alexa Fluor 555 phalloidin (ThermoFisher Scientific). The rabbit anti-NCBP2 antibody was obtained from Sigma and the rabbit anti-NKRF antibody from Life Technology. The rabbit anti-ribophorin I polyclonal antibody was a kind gift from Ewin Ivessa (Center of Biomedical Chemistry, University of Vienna). A goat anti-human IL29 (IFN-λ1) and a goat anti-human IL28A (IFN-λ2), both from Biotechne were used in neutralization assay. Goat anti-rabbit or -mouse immunoglobulins conjugated to Alexa Fluor 488 or Alexa Fluor 555 were purchased from Invitrogen.

### Viral infection for infectious titer determination

Infectious particles present in virus stocks were titrated on Vero E6 cells, according to Barriga et al [41], performing intracellular fluorescence at 48h-72h post infection. In brief, cells seeded at 1.5x10^4^ cells in 24-well plates, were adhered on glass coverslips for 20-24h, then incubated with 150 μL of different dilutions of virus supernatant for 1 h before addition of 1 mL of medium for further incubation. The percentage of infected Vero E6 cells was calculated by counting the cells positively stained by the A1C5 monoclonal antibody, specific to an epitope expressed by N protein of PUUV-CG18-20 strain, and conserved at the N terminus of the N protein of the different orthohantavirus strains used here. The percentage of N protein positive cells (N+) was recorded as a function of the dilution of the supernatants as illustrated in S1 Fig. The viral titers in infectious units per mL (IU/mL) were then calculated from the linear part of the curve as follow:

viral titer = (N x IC) / V x 100, by taking into account the number of plated cells (N), the % of infected cells (IC), the volume of virus (V) in mL. Of note, viruses replicated differently in VeroE6 cells, with titers being around 1–2.5x10^5^ IU/mL for PUUV, 2-5x10^6^ IU/mL for PHV and 5x10^7^- 10^8^ IU/mL for TULV. The indicated MOI used in infection experiments was calculated from the infectious titers of the viral stocks.

For the IFN-λ neutralization assay, orthohantaviruses were pre-incubated with the goat anti-IFNλ1 or λ2 antibodies at 10 μg/mL for 30 min at room temperature according to Prescott et al. [42], then 150 μL of pre-treated viruses were incubated at 37°C for 1h with cell cultures. The viral input was discarded before addition of 1mL of medium/5% FBS. The effect of anti-IFNλ1/λ2 antibodies on virus infectivity was determined at dpi 3.

### Intracellular and membrane immunofluorescence labeling

Cells were plated on glass coverslip (Marienfeld) in 24-well plates at 2x10^4^ cells per well, then, N-protein transfected cells or orthohantavirus-infected cells were treated at the indicated times. For the detection of intracellular components, cells were fixed for 15 min at room temperature with 3.7% formaldehyde (FA, Sigma-Aldrich). Fixed cells were blocked with glycine at 20 mM (Sigma-Aldrich) in phosphate buffered saline (PBS) for 15 min. Cells were permeabilized with 0.5% Triton X100 in PBS for 5 min and then washed with PBS + 0.05% Tween20 (PBS-T). Cells were then incubated with the primary antibodies diluted in PBS-T containing 1% bovine serum albumin (BSA, Cell Signaling), for 1 h. After washing in PBS-T, primary antibodies were detected with Ig species-specific secondary antibodies conjugated either to Alexa Fluor 488 or Alexa Fluor 555 dyes (ThermoFisher Scientific) diluted in PBS-T-BSA 1%, for 1 h. Cells were washed with PBS-T and mounted in Fluoromount DAPI-G (Southern Biotechnology).

In order to detect cell surface antigens, living cells were incubated with primary and thereafter secondary antibodies, both diluted in PBS containing 0.1% sodium azide and complemented with 2% FBS and washings were also performed in PBS with 0.1% sodium azide. Cells were fixed at the end of the reactions with PBS /FA 3.7%, then processed as above for immunostaining.

### Fluorescence staining of recycling endosomes and lipid droplets

Vero E6 cells, seeded and infected as described above, were stained at dpi 3, using Cy3-coupled transferrin (Cy3-Tf, kindly provided by N. Sauvonnet, Institut Pasteur) or a fluorescent marker, 4,4-difluoro-5-(2-thienyl)-4-bora-3a,4a-diaza-s-indacene-3-dodecanoic acid (BODIPY 558/568 C12, ThermoFisher Scientific), to reveal recycling endosomes and lipid droplets, respectively.

To enrich cells in recycling endosomes, infected cells were washed with DMEM then treated with brefeldin A (BFA, ThermoFisher Scientific) diluted to a final concentration of 10 μg/mL. BFA disrupts the trans-Golgi network and blocks recycling vesicles, enhancing their visualization after staining. Cells were incubated with the drug for 30 min at 37°C. Cy3-Tf at a concentration of 1.5 μg/mL in DMEM was then incubated with living cells for 30 min. After washing with PBS, cells were fixed for immunostaining of viral-N proteins.

For lipid droplet detection, infected cells were washed with DMEM and 150 μL of BODIPY 558/568 C12 diluted in DMEM, at a final concentration of 0.1 μg/mL, were added to each well. Cells were incubated with this solution for 10h at 37°C, washed with PBS and then fixed for immunostaining of viral-N proteins.

### Detection of viral RNA by *in situ* hybridization

Infected cells were fixed, permeabilized and then incubated with primary and secondary antibodies as described above for immunofluorescence staining of N protein. Cells were then treated for 5 min at -20°C with 100% of cold ethanol, washed once with PBS and dehydrated in 70% RNase-free ethanol overnight at 4°C. The next day, cells were rehydrated in saline sodium citrate (SSC) buffer at 0.3 M sodium chloride and 30 mM sodium citrate (Invitrogen) for 1 h at room temperature before incubation for 1 h at 60°C in hybridization buffer containing 50% formamide, 10% dextran sodium sulfate salt (Fluka) and 20 μg/mL of salmon sperm DNA (Invitrogen) diluted in SSC. One hundred pmol of a single stranded DNA probe (5’-ACATCAAGGACATTTCCATATCGAAGGCTTGATCTCTCCTT-3’), tagged with Alexa Fluor 488 at its 5’ end, were then added to the hybridization buffer for 5 min at 60°C followed by 4 h at 37°C to detect viral antigenomic (+)RNA and a DNA probe (5’-ACTTATATATATGCACGTAGCATATATATAAGT-3’) tagged with Alexa Fluor 555 (Sigma Aldrich) complementary to the viral genomic (-)RNA. Cells were washed three times with SSC pre-heated at 40°C and then mounted on microscopy slides as described above.

### Quantification of viral genome copy numbers in lysates and supernatants of infected cells

Vero E6, HuH7 and MyglaSWRecB cells were plated at a density of 2.5x10^4^ in 24-well plates and infected the next day at a MOI of 1. Infection and viral RNA quantification within cells and in the supernatant were performed as described [30]. Briefly, cells were incubated for 1 h at 37°C with 150 μL of virus diluted in DMEM/5% FBS, then 1 mL of the same medium was added for further incubation for 3 or 7 days. Recovered supernatants were centrifugated at 1500 rpm for 5 min and kept frozen at -80°C. In parallel, cell layers were lysed in NET/1% TX-100 (150 mM NaCl, 5 mM EDTA, 50 mM Tris-HCl pH 7.5, 0.5 mM with 1% Triton X100) supplemented with a cocktail of protease inhibitor (cOmplete, Roche), and of phosphatase inhibitors (PhosSTOP, Sigma). The post nuclear cytoplasmic fraction was recovered by centrifugation of the cell lysates at 13,000 rpm for 15 min at 4° C and stored at -80°C. RNA from both lysates and supernatants was extracted using QIAmp Viral RNA mini kit (Qiagen) and quantified using a Nanodrop spectrophotometer (ND1000, ThermoFisher Scientific). Reverse-transcription of viral RNA was performed using SuperScript III Reverse Transcriptase (Invitrogen) with virus-specific S segment primers for 1 h at 55°C: 5’-ACCCGCCATGAACAGCAAC-3’ for PUUV, 5’-ACCCGCCATGAACAGCAAA-3’ for TULV and 5’-ACTCGCCATGAGCAGCAGC-3’ for PHV.

To quantify the copy number of viral genomes, internal standard curves specific of each virus were set up and included to all qPCR runs. Short sequences corresponding to viral S segments (nucleotides 73–295) of each virus were inserted into the pSP72 vector (Promega). Reactions for *in vitro* transcription were carried out following manufacturer’s instruction and led to the synthesis of *in vitro* transcribed viral RNA by use of the T7 polymerase of Riboprobe Combination SP6/T7 kit (Promega). Plasmid DNA was then removed by DNaseI treatment (Promega). RNA quantification and residual DNA contamination were determined using a Qubit fluorimeter (ThermoFisher Scientific). Standard curves were determined by serial dilution of *in vitro* transcribed RNA combined to RT-qPCR (see below transcriptomic section) using primers amplifying an S–segment specific internal sequence (Table 1).

### RNA extraction and quantification of viral and cellular genes by RT-qPCR

RT-qPCR assays were performed as previously described [30]. In brief, 5×10^4^ cells seeded in 12-well plates were infected 24 h later at a MOI of 1, with virus diluted in DMEM/5% FBS. RNA from lysates of infected cells was recovered at different time points using TRI Reagent (Sigma), according to the manufacturer’s instructions. RNA from cells treated for 24 h with poly-IC at 10μg/mL was used as a control of IFNs activation. RNAs were quantified with a Nanodrop spectrophotometer (ND1000, ThermoFisher Scientific) and then stored at −80°C. After treatment of the samples with DNase, reverse-transcription was performed using High Capacity cDNA Transcription kit (Applied Biosystems) with random primers. The reactions were carried out in a thermocycler as follows: 10 min at 25°C, 2 h at 37°C, 5 min at 85°C. Quantification of cDNAs was then performed by RT-qPCR using SYBR Green technology (EurobioGreen Mix qPCR 2X Lo-Rox, Eurobio) with gene-specific primers. Primers for amplification of human mRNAs (Table 1) were either devised from online website PrimerBank-MGA-PGA (https://pga.mgh.harvard.edu/primerbank/) or manually designed.

**Table 1:** Sequences of the primers used for RT-qPCR.

Species	Target	Forward sequence (5’-3’)	Reverse sequence (5’-3’)
**Human**	ACTB	AGCCTCGCCTTTGCCGATCC	ACATGCCGGAGCCGTTGTCG
	IFNα (pan)	GTGARGAAATACTTSCAAAGAATCAC	TCTCATGATTTCTGCTCTGACAA
	IFNβ	GTCTCCTCCAAATTGCTCTC	ACAGGAGCTTCTGACACTGA
	IFNλ1	TCCTAGACCAGCCCCTTCA	GTGGGCTGAGGCTGATA
	IFNλ2/3	CTGACGCTGAAGGTTCTGGAG	GATATGGTGCAGGGTGTGAAGG
	RSAD2	TTGGACATTCTCGCTATCTCCT	AGTGCTTTGATCTGTTCCGTC
	XAF1	GCTCCACGAGTCCTACTGTG	GTTCACTGCGACAGACATCTC
**Bank vole**	Actb	GGAAATCGTGCGTGACATCA	CTCGTAGCTCTTCTCCAGGG
	Ednrb	CGGCCATCTTTTACACCCTG	TTTGGCCACTTCTCGTCTCT
	Gipr	CGAAGTCAAAGCCATTTGGT	ATGGGAAATCCCTGAACACA
	Ifit1	CAGGCCTACGTGAGACACAA	CTTCTCTTGCCCAACTGCTC
	Ifnα	GCCTCAGACTCATAACCTCAGG	GTCCTTTCYGTCCTTCAGGC
	Ifnβ	CTCTCCATAACCCTGTCCATCAAC	GTCTTTCTTCTCCATCTGTCCTG
	Ifnλ2/3	TCAGATGCAAAGGAATGTCACC	CACTCTTCTATGGCGTCCTTGG
	Iigp1	GCAATCAGCGTGATGAAGAA	GATCTGATGCAGGACCGTTT
	Irgm1	CAAATTTGGTGTCGACGATG	TATGCAGGGTTTGGGACTTC
	Mpeg1	GCCCAACTTCAGAACTCAGC	GACCAACAGTTCTGCCAGGT
	Msr1	AGCTGGCATCCCTGGAAATA	TTGATATGCTTGCACTGCCC
	Mx1	GCCATCAACAAAGCCCAGAA	CAGGTCAATCAGGGTCAGGT
	Rsad2	CTGTGAGCATCGTGAGCAAT	CGCAGAAGTCAGCATACGAG
	Xaf1	GACCAAGTCTGAGGAAGGGA	TGGGCATTGTTTCATGTGGG
**PUUV**	N	AGGATGCAGAGAGAGCAGTG	TGCCATCCTTCTCTTGTAGTC
**TULV**	N	AAGATGCAGAAAAGACGGTGGA	TGCAAGCTGCCTCTTGAAGTC
**PHV**	N	AGGAAGCTGAACGGACGGTG	TGCAAGCTGCCTCTTGAACTC

In designing primers for amplification of mRNA from the bank vole cell line, transcripts corresponding to putative *M*. *glareolus* orthologs of mouse IFNα, β and λ2/λ3 genes were screened and found by BLAST [43] in a recently published, unannotated bank vole transcriptome [44]. Data were normalized to the actin mRNA and presented as relative expression compared to non-infected cells (2^-ΔΔCt^).

### Electron microscopy analysis

To visualize the cellular compartments following infection, as well as the presence of viral particles both inside the cells and in the extracellular space, cellular pellets were prepared as follows for EM analysis. Vero E6, HuH7 and MyglaSWRecB cells were seeded in culture flasks and infected 24 h later with PUUV, TULV or PHV at a MOI of 2. After 5 days, cells were washed in PBS before being recovered by trypsinization, centrifuged for 5 min at 1000 rpm, then pelleted cells were suspended in a fixation buffer (4% FA, 1% glutaraldehyde, pH 7.3). Pellets were kept at least 48 h at 4°C and then a few days at room temperature before being treated for EM.

Samples were washed in PBS and post-fixed by incubation for 1 h with 2% osmium tetroxide (Agar Scientific). Cells were then fully dehydrated in a graded series of ethanol solutions and propylene oxide. They were impregnated with a 1∶1 mixture of propylene oxide/Epon resin (Sigma) and left overnight in pure resin. Samples were then embedded in Epon resin (Sigma), which was allowed to polymerize for 48 hours at 60°C. Ultra-thin sections (90 nm) of these blocks were obtained with a Leica EM UC7 ultramicrotome (Leica Microsystem). Sections were stained with 2% uranyl acetate (Agar Scientific), 5% lead citrate (Sigma), and observations were made with a transmission electron microscope (JEOL 1011).

### Pull down of cellular proteins in complex with viral nucleocapsids for mass spectrometry analysis (LC/MS/MS)

#### Sample preparation

Transfection, pull down and LC/MS/MS were performed on sample triplicates, as previously described [30]. In brief, 10^6^ HEK293T cells were transfected, using the JetPRIME reagent (Polyplus), with 1 μg of plasmid pCiNeo-streptag encoding PUUV-N, TULV-N or PHV-N or pCiNeo-streptag empty plasmid as a control, and incubated for 24 h at 37°C. For each condition, 1 mg of proteins was pulled down for 1 h, at 4°C, on 25 μL of packed sepharose beads linked to Strep-Tactin (IBA). After washing in lysis buffer (NET/1% TX100), the beads were suspended in 25 mM NH_4_HCO_3_, pelleted by centrifugation and kept on ice covered by a film of bicarbonate ready to be processed for mass spectrometry analysis.

#### Peptide identification by LC-MS/MS

Proteins on beads in association with viral N protein of PUUV, TULV or PHV, or beads which have been incubated with the lysate of cells transfected with the empty plasmid as a control, were incubated overnight at 37°C with 20 μL of trypsin (sequencing grade, Promega) at 25 μg/mL in 25 mM NH_4_HCO_3_. Peptides were desalted using ZipTip μ-C18 Pipette Tips (Millipore). Peptide mixtures were analyzed by a Q-Exactive Plus coupled to a Nano-LC Proxeon 1000 both from ThermoFisher Scientific. Peptides were separated by chromatography with the following settings: Acclaim PepMap100 C18 pre-column (0.075 x 20 mm, 3 μm, 100 Å), Pepmap-RSLC Proxeon C18 column (0.075 x 500 mm, 2 μm, 100 Å), 300 nl/min flow rate, a 98 min acetonitrile (ACN) gradient from 95% solvent A (H_2_O/0.1% FA) to 35% solvent B (100% ACN/0.1% FA) followed by column regeneration, giving a total acquisition time of 145 minutes. Peptides were analyzed in the Orbitrap cell in positive mode, at a resolution of 70,000, with a mass range of *m/z* 200–2000 and an AGC target of 3.10^6^. MS/MS data were acquired in the Orbitrap cell in a Top20 mode. Peptides were selected for fragmentation by Higher-energy C-trap Dissociation (HCD) with a Normalized Collisional Energy of 27%, a dynamic exclusion of 60 seconds, a quadrupole isolation window of 1.4 Da and an AGC target of 2.10^5^. Peptides with unassigned charge states or monocharged were excluded from the MS/MS acquisition. The maximum ion accumulation times were set to 50 msec for MS and 45 msec for MS/MS acquisitions.

### Quantification of protein abundance variation

MS raw files were processed using PEAKS Online X (build 1.5, Bioinformatics Solutions Inc.). Data were searched against the Human Uniprot release 2021_03 database consisting of reviewed-only sequences including 20387 total entries. The sequences of hantaviral N proteins were added for the search, as internal control. Parent mass tolerance was set to 20 ppm, with fragment mass tolerance of 0.05 Da. Specific tryptic cleavage was selected and a maximum of 2 missed cleavages was authorized. For identification, the following post-translational modifications were included: acetyl (Protein N-term), oxidation (M), deamidation (NQ) as variables and half of a disulfide bridge (C) as fixed. Identifications were filtered based on a 1% FDR (False Discovery Rate) threshold at both peptide and protein group levels. Label free quantification was performed using the PEAKS Online X quantification module, allowing a mass tolerance of 20 ppm for match between runs and auto-detection of retention time shift tolerance. Protein abundance was inferred using the top 3-peptide method and TIC was used for normalization. Multivariate statistics on protein measurements were performed using Qlucore Omics Explorer 3.7 (Qlucore AB, Lund, Sweden). A two-group comparison was used to compare sequentially proteins identified in each viral N protein samples and in the empty plasmid control sample, a p-value lower than 0.05 was used to filter differential candidates considered as potential N protein interaction partners. The PRIDE database, dedicated to mass spectrometry-based proteomics data [45], was chosen to deposit our LC/MS/MS data.

### Transcriptomic analysis of bank vole cells upon infection with PUUV or PHV

#### RNA preparation from infected cells

MyglaSWRecB cells were plated at 2.5x10^5^ cells per well of 12-well microplates and infected 24h later. Four wells per condition were incubated either with PUUV or PHV at MOI of 0.5 or remained non-infected. RNA was prepared from cells at dpi 5. Cells were washed with PBS and incubated for 5 min at room temperature with 500 μL of Trizol (Tri Reagent, Sigma Aldrich) before addition of 150 μL of chloroform for 10 min and centrifugation at 4°C for 10 min at 11,000 rpm. The aqueous phase was recovered and precipitated with isopropanol. After centrifugation, the pellet was washed in 70% ethanol and then suspended in 50 μL of RNase free water.

#### RNA sequencing and transcriptomics

RNA preparation was used to construct strand-specific single-end cDNA libraries according to the manufacturer’s instructions (TruSeq Stranded mRNA sample prep kit, Illumina). Illumina NextSeq 500 sequencer was used to sequence libraries. The RNA-seq analysis was performed with the Sequana framework [46]. First, the viral infection has been verified using the mapper pipeline and specific viral RNA sequences are detected in the infected samples. Then, we used the RNA-seq pipeline (v0.13.0), which is built on top of Snakemake 5.8.1 [47] and is available online (https://github.com/sequana/sequana_rnaseq). Reads were trimmed from adapters using Cutadapt 2.10 [48] and then mapped to the *M*. *musculus* GRCm38/mm10 genome and *M*. *glareolus* draft genome using STAR 2.7.3a [49]. Sequencing of the bank vole genome was recently performed using a combination of shotgun, Chicago, and Dovetail HiC library reads [50]. The assembly yielded a total of over 4300 scaffolds, 39 of which were larger than 50 Kb and covered 99% of the genome, and was annotated with the GAWN pipeline (https://github.com/enormandeau/gawn) using a BLASTX search [43] against the Swissprot database (UniProt Consortium 2019). FeatureCounts 2.0.0 [51] was used to create the count matrix, assigning reads to features with strand-specificity information. Quality control statistics were summarized using MultiQC 1.8 [52]. Statistical analysis on the count matrix was performed to identify differentially regulated genes by comparing infected to non-infected samples. Clustering of transcriptomic profiles was assessed using a principal component analysis (PCA). Tests for differential expression were performed using DESeq2 library 1.24.0 [53] scripts based on SARTools 1.7.0 [54] indicating the significance (Benjamini–Hochberg-adjusted P-values, FDR < 0.05) and the effect size (fold change) for each comparison. Finally, enrichment analysis was performed using modules from Sequana. The GO enrichment module uses the PantherDB [55] and QuickGO [56] services; the KEGG pathways enrichment uses the gseapy (https://github.com/zqfang/GSEApy/), EnrichR [57], KEGG [58], and BioMart services. All programmatic access to the online web services was performed via BioServices [59].

### Validation of differentially expressed genes by RT-qPCR

To evaluate the reliability and reproducibility of the results obtained in the RNA-Seq analyses, we used RT-qPCR to validate the gene expression patterns of selected genes. Among the differentially expressed genes (DEG) of bank vole identified by RNA-Seq, 11 genes were selected to be validated by RT-qPCR. To design primers for DEG, which could only be detected by using the mouse reference genome, transcripts of putative bank vole orthologs were identified in the bank vole transcriptome [44] as described above.

### Statistical analysis

Groups of data are represented as the mean of biological triplicates and the standard deviation to the mean was calculated. For RT-qPCR analysis of cellular gene expression, the mean of each virus infected cells was compared to the mean of non-infected cells by standard One-way ANOVA using Dunnett method for multiple comparisons. For quantification of viral genome copies in lysates and supernatants, as well as for infections calculated as the percentage of N+ cells, standard Two-way ANOVA using Šidák method for multiple comparisons was applied. Finally, statistical analysis of neutralization assay was performed using standard Two-way ANOVA with Tukey method. Only data with 95% confidence interval were considered significant with * p< 0.0332, ** p<0.021, *** p<0.0002 and **** p<0.0001, while “ns” indicates non-significant variation.

## Results

### Susceptibility to infection of human and rodent host-derived cell lines differs with orthohantavirus species

**Fig 1:**
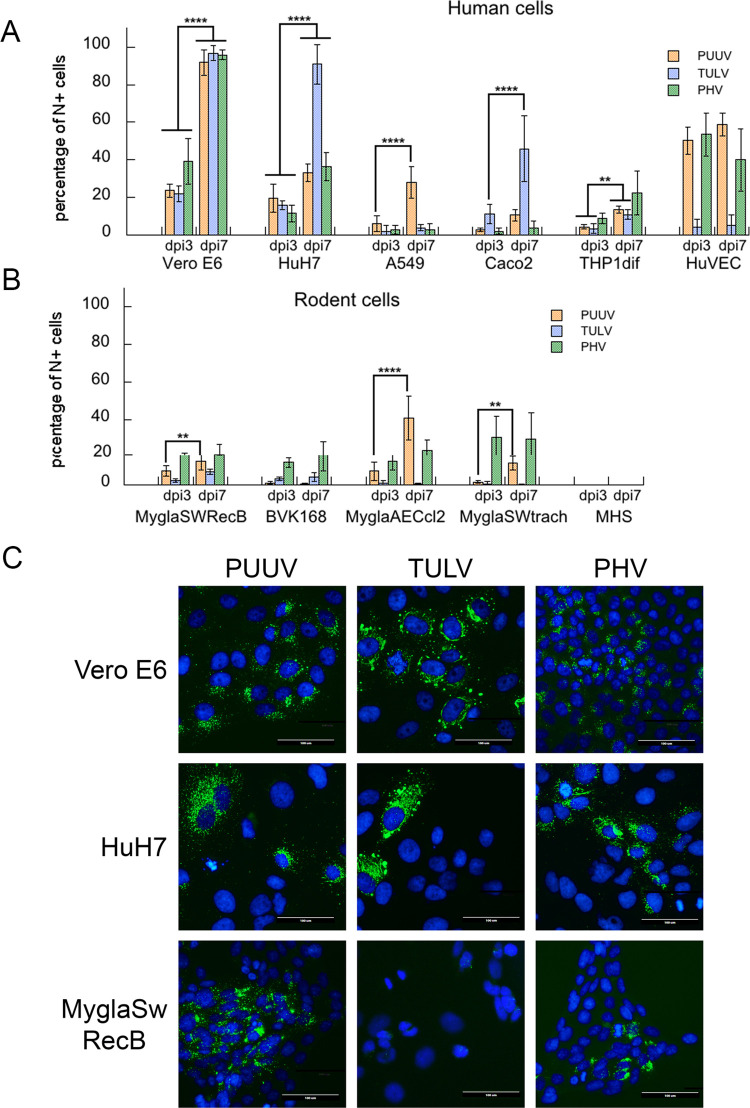
Susceptibility of human and rodent cells to orthohantavirus infection. Cells grown on glass coverslips were inoculated with a MOI of 1 with the different orthohantaviruses, then used at dpi3 and dpi7 for intracellular immunofluorescence staining of the viral N protein using the A1C5 monoclonal antibody to evaluate the infectivity of PUUV (orange bars), TULV (blue bars), and PHV (green bars). The histogram in (A) shows the percentage of infected cells (N+) of human origin derived from liver (HuH7), lung (A549), intestine (Caco2), as well as differentiated monocytes (THP1dif) and vascular endothelial cells (HUVEC). The non-human primate Vero E6 cells used to prepare virus stocks were added as a control. Histogram in (B) shows the susceptibility of rodent cell lines to orthohantaviruses, including renal cells (MyglaSWRecB and BVK168) and airway epithelia (MyglaSWTrach and MyglaAECcl2) from bank vole. The results were obtained from at least three independent experiments and the error bars correspond to standard deviation to the mean. Statistical analysis between dpi3 and dpi7 was performed using Two-way ANOVA and are shown according to p-values represented by *p-value <0.0332, **p-value<0.0021, ***p-value<0.0002, ****p-value<0.0001. Non-significant variations are not marked and are further detailed in S1 Fig. Pictures in (C) show N immunofluorescence detection at dpi 3 in Vero E6, HuH7 and MyglaSWRecB cells infected by PUUV, TULV or PHV. Viral N protein is stained in green and the nucleus in blue with DAPI (4′,6-diamidino-2-phenylindole). The scale bar corresponds to 100 μm.

In order to provide cellular models to study orthohantavirus interactions with their hosts, we first compared the permissiveness of epithelial and endothelial cell lines derived from different organs of human or rodent origin known to be targeted by orthohantaviruses, i.e. lung, kidney, macrophages, as well as additional cell types. In order to determine if cells could be infected by pathogenic PUUV, low-pathogenic TULV and non-pathogenic PHV, using a MOI of 1, we quantified by immunostaining the percentage of infected cells expressing the viral N protein at dpi 3 and 7 (Fig 1). These two time points were chosen since orthohantaviruses replicate slowly, allowing then the visualization of the N protein of PUUV and PHV at day 3 (dpi 2 for TULV) to titrate viruses, while day 7 post infection corresponded to the time used for the preparation of viral stocks and to evaluate whether the virus was amplified. Simian Vero E6 cells, which are susceptible to infection, due to the lack of expression of type-I IFN, were used to produce all three viral stocks and as a reference. As shown in the histogram in Fig 1A, the sole human cell lines to be infected by the three orthohantaviruses were the hepatocyte HuH7 cell line, for which the percentage of N+ cells increased through time reaching 33%, 90% and 36% for PUUV, TULV and PHV, respectively, and the THP1 cell line differentiated into macrophages, reaching, at dpi 7, 10 to 20% of infection depending on the virus. At this late time point of infection (dpi 7), the lung alveolar epithelial A549 cells were susceptible to PUUV (30% of N+ cells), while TULV and PHV could barely infect them (less than 5% of N+ cells). In contrast, the intestinal epithelial cell line, Caco-2, was mainly susceptible to TULV, reaching 75% of infected cells, while PUUV and PHV at most, gave rise to 10% of N+ cells. Interestingly, around 50% of the HUVEC were infected by PUUV and PHV but this percentage did not increase with time and these cells were scarcely susceptible to TULV infection.

In parallel, we evaluated the susceptibility to orthohantavirus infection of cell lines derived from the animal reservoir of PUUV (Fig 1B). Four cell lines have been derived from lung (MyglaAECcl2 and MyglaSWTrach) or kidney (MyglaSWRecB and BVK) of the bank vole, the natural host of PUUV. Regardless of whether they originated from the respiratory tract or the kidney, bank vole cell lines could be infected with PUUV and PHV. However, BVK168 cells were poorly infected by PUUV, while in similar conditions–at dpi7- around 30% of MyglaSWRecB cells and 40% of MyglaAECcl2 cells were found to be N+. Infection of MyglaSWTrach cells was obtained but was higher with PHV (30%) than with PUUV (10%). Importantly, TULV infection was not successful in these *M*. *glareolus* cell lines. A *M*. *musculus* derived cell line, MHS, was not infected by any of the three orthohantaviruses. Remarkably, statistical analysis revealed that the N+ proportion of the different Mygla cells only increased significantly from dpi3 to dpi7 for PUUV.

Our results show that, even using an *in vitro* model of infection, orthohantaviruses exhibit differences in cell specificity depending on the cell type and the host from which they originated.

### Bank vole cells, in contrast to HuH7 cells, release infectious PUUV particles

**Fig 2:**
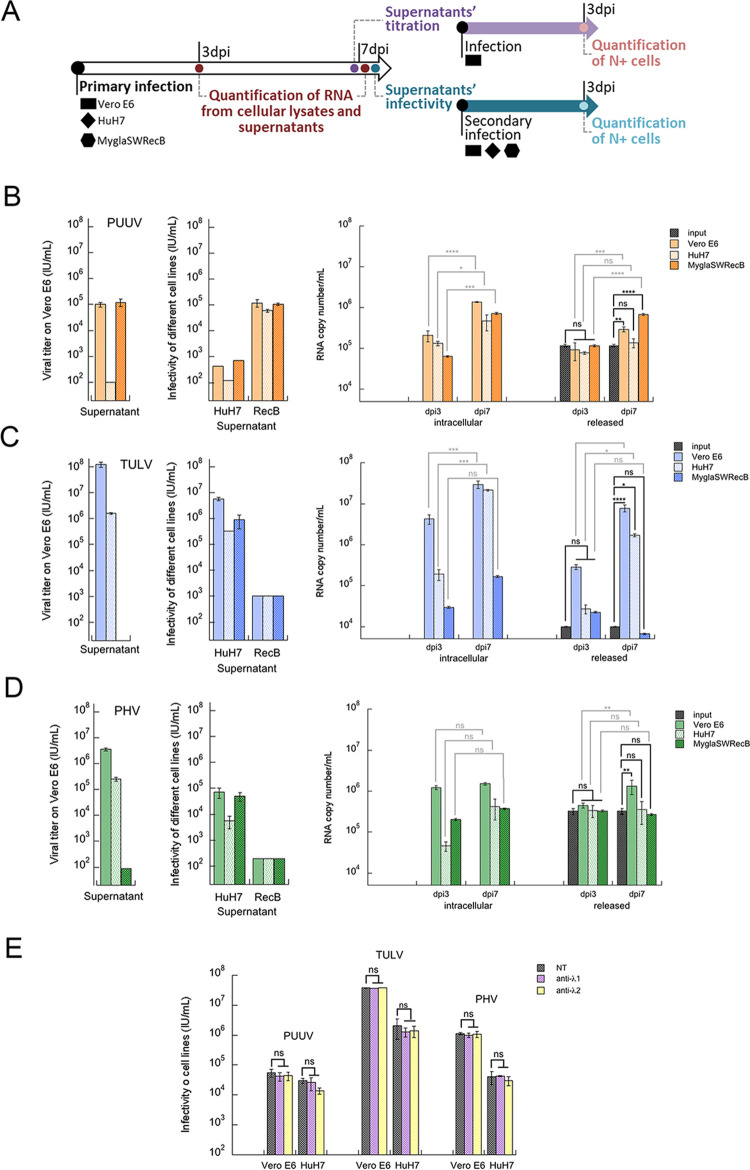
Ability of human and bank vole cells to produce infectious viruses. A schematic representation of the workflow of experiments performed to evaluate the capacity of production of infectious virus by the cell lines further used in the study is shown in (A). Supernatants of Vero E6, HuH7 and MyglaSWRecB cells primarily infected (1^st^) at MOI 0.5 by PUUV (B), TULV (C) or PHV (D) were recovered at dpi 7 for determination of their infectious titers on Vero E6 cells (left panels) and then for their ability to secondary (2^nd^) infect HuH7 and MyglaSWRecB cells (middle panels). Infectious titers were calculated from the percentage of N+ cells determined by immunofluorescence staining at dpi 3 as described in the Materials and Methods section. In parallel, copies of viral RNA produced in the three cell lines infected by PUUV (B), TULV (C) and PHV (D) were quantified at dpi 3 and dpi 7 (right panels) both, from the cellular fraction (intracellular viral RNA) and from their supernatants (released viral particles). The histograms show the copy numbers of viral RNA per sample calculated based on RNA standard curves corresponding to S segments specific of each orthohantavirus. The same viral preparation was used for each virus to infect the different cell lines (input). The error bars represent the standard deviation to the mean of technical replicate of biological triplicate for each sample. In (E), an IFN-λ neutralization assay was performed. PUUV, TULV or PHV were either left untreated (NT) or were pretreated with anti-human IFN-λ1 or with anti-human IFN-λ2 antibodies prior to infection at a MOI of 0.5, then their infectious titers on Vero E6 and HuH7 human cells were compared at dpi 3. Statistical analyses were performed to compare the amount of RNA between day 3 and day 7 in the cell lysates, as well as the amount of RNA among day 3, day 7 and the input in the supernatant. For neutralization assay, infection in absence of neutralizing antibodies was compared to those in presence of either anti-IFNλ1 or anti-IFNλ2. “ns” indicates not significant differences, while the stars (*) mark significant variations as explained in Fig 1. RecB was used as an abbreviation of MyglaSWRecB in the middle B-D panels.

Cell susceptibility analyses (Fig 1) revealed differences in entry and replication of orthohantaviruses in different cell lines. To further investigate these results, we compared the viral life cycle of PUUV, TULV and PHV in human and bank vole cells as compared to permissive Vero E6 cells by looking whether infection led to production of mature infectious particles. A schematic representation of the procedure used is shown in Fig 2A. We chose the human HuH7 cell line, which could be infected by the three orthohantaviruses, and the rodent MyglaSWRecB cell line, which proved to be a suitable model exhibiting a species-specific restriction of TULV infection, while being efficiently infected with PUUV and PHV. We first determined infectious titers on Vero E6 cells of the human and bank vole cell supernatants, recovered at dpi 7 following infection by each one of the three viruses as compared to Vero E6 cells (Fig 2B–2D, left panels, and S1A-C, right panels). The supernatants of HuH7 and MyglaSWRecB infected cells were also tested in secondary infections on these same human and rodent cell lines by evaluating the percentage of N+ cells at dpi 3 (Fig 2B–2D middle panels) and extrapolating an infectious titer. In order to compare the infectivity of the supernatants, the same dilutions were used in primary and secondary infections for each orthohantavirus.

While PUUV efficiently infected and produced infectious particles in Vero E6 cells with a titer of 10^5^ IU/mL, its titer in infected HuH7 cells (Fig 2B left panel) was at the limit of detection (<10^2^ IU/mL) and as a result did not infect the three cell lines. Interestingly MyglaSWRecB cells infected with PUUV produced infectious particles at similar level to Vero E6 with a viral titer of 1.2x10^5^ IU/mL and could re-infect these three cell lines (middle panel).

Concerning TULV, high viral titers were produced in the supernatant of infected Vero E6 cells (10^8^ IU/mL) and TULV was infectious for HuH7 cells, but not for MyglaSWRecB cells (Figs 2C, left panel and S1), consistent with the host specificity of bank voles for PUUV and not TULV. However, although HuH7 cells were highly susceptible to TULV infection (Fig 1A) the amount of infectious virus produced in HuH7 supernatant was considerably reduced, as compared to Vero E6 cells, with a titer reduction by 2 log (10^6^ IU/mL) and this supernatant was not able to significantly re-infect the same cell line (Fig 2C, middle panel).

In the case of PHV, and similarly to what was observed with TULV the supernatant from PHV infected HuH7 cells was less infectious than the one from Vero E6 with a viral titer of 2x10^5^ IU/mL versus 2x10^6^ IU/mL in Vero E6 (Fig 2D, left panel). Moreover, this supernatant was barely infectious when used in secondary infection of HuH7 cells. Surprisingly, although MyglaSWRecB cells were susceptible to PHV infection (Fig 1B) its supernatant contained almost no infectious particles exhibiting a titer of 10^2^ IU/mL as compared to 2x10^6^ IU/mL in PHV-infected Vero E6 cells. In addition, this bank vole cells’ supernatant was not able to re-infect the three cell lines (Fig 2D, middle panel).

The differences in the production of infectious particles, depending on the cell lines and virus species, highlight the different levels of cellular restriction to the achievement of the hantaviral cycles, complementing the results on the susceptibility of cells to infection. Importantly, these results altogether show that the production of infectious particles on human HuH7 cells was restricted for all three viruses, whereas three distinct outcomes were observed on bank vole cells: production of infectious PUUV particles, restriction at an early stage of TULV infection, and restriction at a final stage impairing PHV particle egress.

### Quantification of viral RNA copy number in infected cells confirms the differences in orthohantavirus production associated with cell susceptibility

Since the supernatants of infected cells exhibited variable infectivity, depending on the orthohantavirus used, and to validate the above observations, we tested whether viral genomes were replicating intracellularly and could be detected in the supernatant, reflecting the release of viral particles. Indeed, it could be that viral particles would be produced without being infectious due to the production of immature virions, or due to the presence in the supernatant of cytokines with antiviral activity. In this regard the virus stocks produced on Vero E6 could be associated to IFN-λ induced by orthohantavirus infection as described by Prescott et al. [42]. In order to test whether the presence of some IFN-λ secreted by Vero E6 cells could impact orthohantavirus infectivity in our experimental conditions, we performed a neutralization assay. Pre-incubation of the three viruses with anti-IFNλ1 or anti-IFNλ2 anitbodies did not significantly impact the viral growth since titers on Vero E6 cells, as well as on human HuH7 and A549 cells were not improving by such treatment (Fig 2E).

Beside the determination of the viral titers produced in the supernatants of HuH7 and MyglaSWRecB cells infected either by PUUV, TULV or PHV as compared to Vero E6 (Fig 2B–2D, left panels, and S1), we quantified by RT-qPCR the number of copies of viral genomes in cell lysates, as well as those released in the cell supernatants, at dpi 3 and dpi 7 (Fig 2A and 2B–2D, right panels). The RNA copy numbers were determined by referring to standard curves of *in vitro* transcribed RNA corresponding to a specific sequence of each viral S segment.

PUUV was replicating from dpi 3 to dpi 7 in Vero E6, HuH7 and MyglaSWRecB cells, as shown by amplification of the amount of intracellular copies of viral genomes (Fig 2B, right panel). In parallel, viral RNA was not present at day 3 in the supernatants, but clearly detectable at dpi 7 in Vero E6 (3x10^5^ copies/mL) and in MyglaSWRecB (6.7x10^5^ copies/mL) cells. Of note, the copy number of viral genomes in the supernatant of HuH7 cells remained low (around 10^5^ copies/mL) and was not statistically significant, not exceeding the viral input.

As expected from the much higher titers of TULV (around 10^8^ IU/mL), as compared to PUUV (around 10^5^ IU/mL), a large and significant number of intracellular copies of TULV genome was measured in Vero E6 (3x10^7^ copies/mL) and HuH7 (2x10^7^ copies/mL) cells at dpi 7 as compared to dpi 3, while viral RNA load did not increase intracellularly and was not detected in MyglaSWRecB cell supernatant (Fig 2C, right panel), confirming that these cells were not permissive to TULV. Besides, similarly to PUUV infected HuH7 cells, a low amount of TULV RNA copies was detected at dpi 7 (1.5x10^6^ copies/mL) in HuH7 supernatant, correlating with its lower infectivity (Fig 2C, left panel) as compared to a higher copy number of TULV genome (7.5x10^6^ copies/mL) found in Vero E6 supernatant.

In the case of PHV (Fig 2D, right panel), the number of RNA copies, which was already high at dpi 3 in Vero E6 cell lysate (1.1x10^6^ copies /mL), did not significantly increase intracellularly at dpi 7 (1.4x10^6^ copies/mL). In contrast, the amount of viral RNA in HuH7 and MyglaSWRecB cells was significantly lower and did not increase either with time from dpi 3 to dpi 7 (around 3x10^5^ copies/mL). Correlating with this low level of replication in HuH7 and MyglaSWRecB cells, the amount of copies of viral RNA released in the supernatant of these two cell lines was low, not exceeding the viral input (around 3x10^5^ copies/mL) at both time points, as compared to the amount of PHV genome (10^6^ copies/mL) in Vero E6 supernatant. This confirms the fact that PHV replication in bank vole cells did not lead to production of infectious viral particles.

### Electron microscopy analysis reveals a peculiarity in the maturation of TULV in human cells and in the localization of PHV in bank vole cells

Our search for infectious particles and quantification of viral RNA highlighted different aspects of orthohantavirus maturation, depending on the virus and the cell line. We next performed EM analyses to determine whether complete viral particles could be detected and released by infected cells. Vero E6, HuH7 and MyglaSWRecB cells were processed for EM at dpi 5.

**Fig 3:**
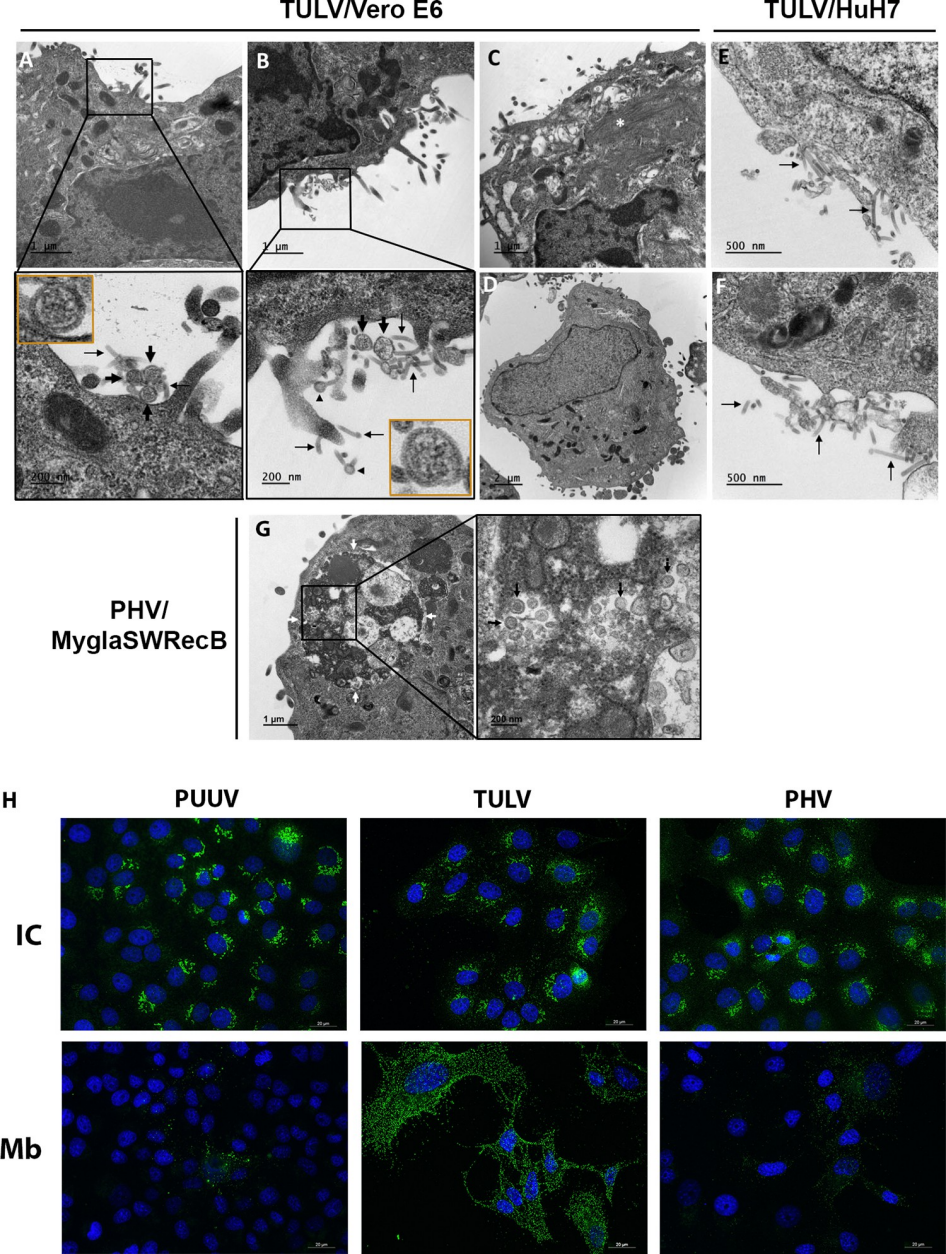
Maturation of viral particles assessed by electron microscopy and immunofluorescence assay. Panels A-G show thin sections of fixed cells processed by transmission EM. Vero E6 cells were either infected with TULV (A-C) or remained non-infected (D). The lower panels in A and B correspond to a magnification of the plasma membrane area framed by a square in the upper panels. Viral particles (insets), at the plasma membrane and in the extracellular space, are marked with large black arrows and tubular structures are indicated with thin black arrows. In (C), the white asterisk highlights the filamentous structure induced by TULV infection. HuH7 cells infected by TULV (E, F) only show tubular structures at the plasma membrane and the extracellular space (thin black arrows). Multivesicular vacuoles containing viral particles (black arrowheads) and their magnification are seen in MyglaSWRecB cells infected by PHV (G). Immunofluorescent staining of membrane (Mb) and intracellular (IC) viral glycoprotein Gn is shown in panel H for Vero E6 cells infected with PUUV, TULV and PHV. The scale bar corresponding to 20 μm indicates the magnification.

It was not possible to observe any PUUV particles by EM, reflecting its low replication rate. However, in the case of TULV, viral particles were identified, at the cell surface of infected Vero E6 cells (large black arrows in Fig 3A and 3B; see in two insets high magnifications of these virions). Interestingly, these viral particles were often associated with tubular structures (thin black arrows in Fig 3A and 3B), which formed in some cases a pan-shaped end (arrowheads in Fig 3B). These tubular structures are most likely of viral origin, as they were not observed in uninfected cells (Fig 3D). Of note, as recently described by others [60], we also observed accumulations of intracellular filaments in infected Vero E6 cells (Fig 3C, white asterisk), not seen in uninfected cells (Fig 3D). Importantly, no mature virions could be visualized at the surface of infected HuH7 cells, but these cells exhibited at their surface the same tubular structures as those found in infected Vero E6 cells (thin black arrows in Fig 3E and 3F). This suggests a defect in TULV maturation in HuH7 cells.

In addition, under condition where at least 50% of cells were infected by PUUV, TULV or PHV, as shown by intracytoplasmic Gn staining (Fig 3H, IC), we observed a striking TULV-Gn staining at the surface of Vero E6 cells, not seen with PUUV or PHV (Fig 3H, Mb). This could be paralleled with the EM observation of empty tubular structures in TULV infected Vero E6 cells, suggesting that it may correspond to membrane areas decorated with viral glycoproteins and protruding from the cell surface.

In the case of PHV, as for PUUV, a low level of particles was produced in Vero E6 cells and virions were only rarely visualized in ultrathin preparations used for EM analysis. However, some intracellular PHV particles were observed in MyglaSWRecB cells. In these cells, the virions (large black arrows in Fig 3G) were found to be present in large intracellular cisterns that probably correspond to autophagic compartments (delimited by white arrows in Fig 3G).

These data are consistent with the fact that HuH7 cells did not produce a large amount of TULV particles in their supernatants and that PHV replicated in the bank vole cell line without releasing infectious particles while accumulating large amounts of viral proteins intracellularly.

### Distinct cellular organizations of PUUV, TULV and PHV N proteins and interaction with human cellular partners suggest different roles for these proteins

To better understand the way viral particles interact with cells during their viral cycles, we investigated by immunofluorescence staining the morphology and subcellular localization of PUUV-N, TULV-N and PHV-N in infected Vero E6 cells. Whether N proteins of the different orthohantaviruses could recruit some specific cellular factors was also evaluated by proteomics analysis of cellular proteins in complex with N proteins, which were expressed after plasmid transfection in human HEK293T cells. In this case the comparison with MyglaSWRecB cells could not be carried out due to a lack of tools for the identification of bank vole proteins.

#### Cellular distribution of the viral nucleocapsids

**Fig 4:**
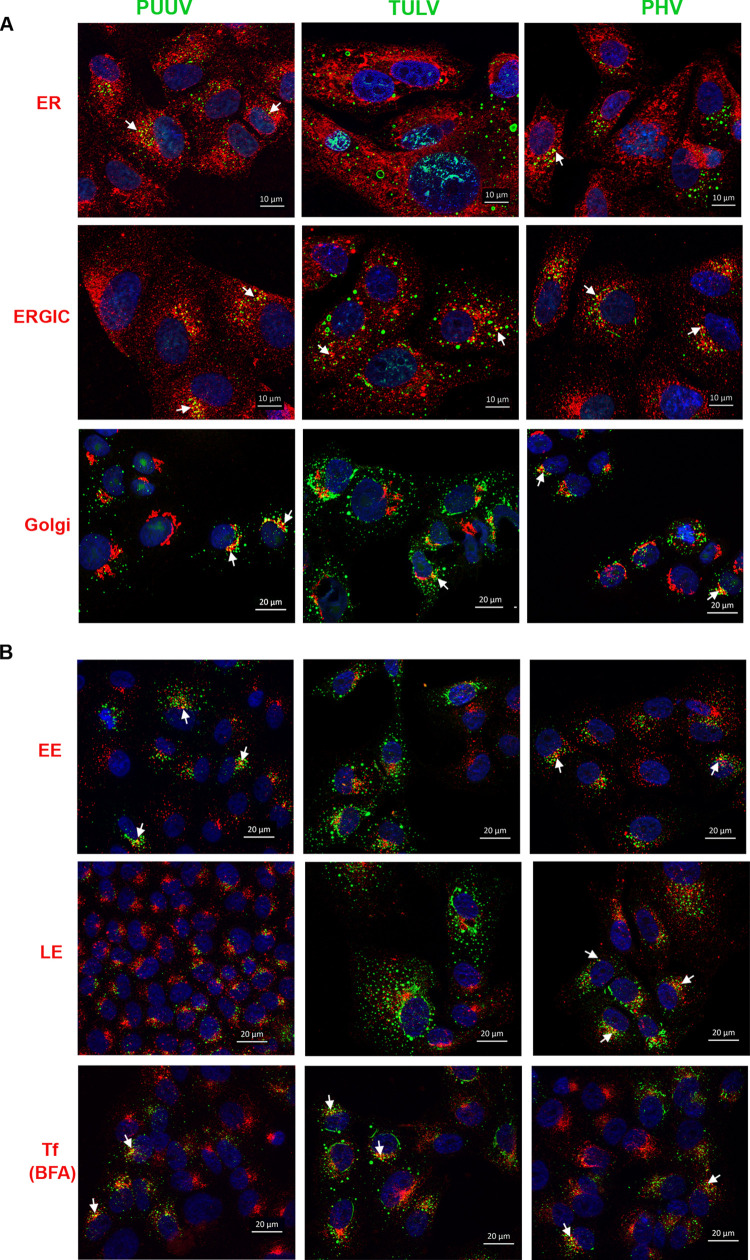
Distribution of N proteins relative to cellular compartments. Vero E6 cells grown on glass coverslip were infected with PUUV, TULV or PHV at MOI 1. Intracellular co-localization appeared in yellow in the merge panels following staining of the N protein in green, and markers of different cell compartments in red. In (A) the co-localization of N with compartments of the secretory pathway (ER, ERGIC and Golgi) is shown, while in (B) the co-localization with the early (EE), late (LE) and recycling endosomes from the endocytic pathway is shown. Recycling endosomes were revealed via transferrin trafficking (Tf) in presence of BFA. DAPI labels nuclei in blue.

Immunofluorescence staining of infected Vero E6 cells highlighted that only a small fraction of N proteins localized nearby early compartments of the secretory pathway, as shown in Fig 4A (see arrows) and S2 Fig. Indeed, a small fraction of PUUV-N and PHV-N co-localized with ER, ERGIC and Golgi. This is consistent with the fact that the N protein interacts with the envelope glycoprotein during viral particle assembly as comforted by a partial co-localization of N and Gn in infected cells (S2 Fig). Of note, the high expression level of TULV-N protein seems to disturb the organization of these cell compartments. Apart from allowing viral entry, the endocytic pathway is known to interact with the secretory pathway and could play a role in orthohantavirus egress [61]. In line with this, the small-sized granules of N proteins were observed in the same area as the early endosomes (EE). A small fraction of PUUV-N and PHV-N was also localizing close to late endosomes (LE), and more importantly in the case of all three viruses, with the recycling compartments, as shown by co-staining with fluorescent transferrin (Figs 4B and S3).

**Fig 5:**
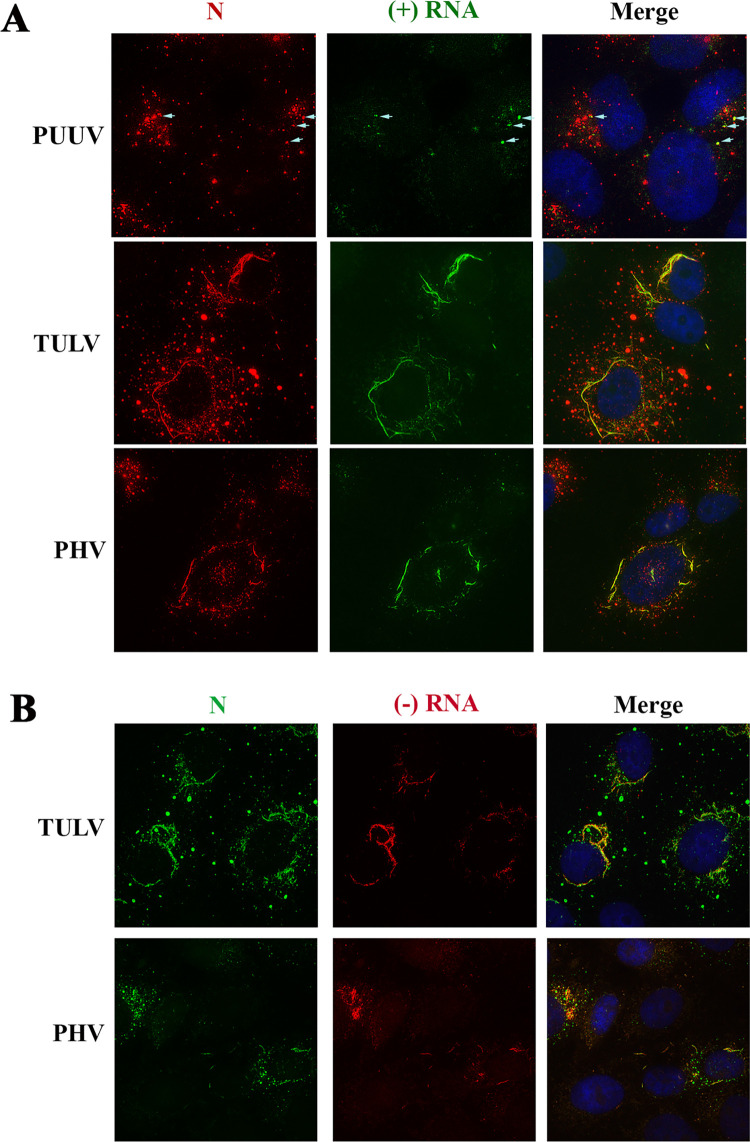
Co-localization of viral RNA with filamentous structures of N protein. Vero E6 cells infected with PUUV, TULV or PHV were treated for *in situ* hybridization by incubation with fluorescent RNA probes either complementary of the viral genomic (-)RNA (green) or of the antigenomic (+)RNA (red). The viral nucleocapsids were detected by immunofluorescence staining with the A1C5 antibody but using secondary antibodies conjugated to Alexa 555 (red) or Alexa 488 (green) according to the fluorescence of the RNA probes. Colocalization of the N filaments with viral RNA appears in yellow in the merge panels. White arrows indicate co-localisation of N dots with PUUV RNA probes.

In addition, by performing concomitant labeling of the viral N protein and the viral genome (or antigenome), using fluorescence conjugated probes, we clearly observed that the N filaments, both in TULV and PHV-infected Vero E6 cells, co-localized with hantaviral RNA of genomic and antigenomic polarity (Fig 5). This does not exclude co-localization of the viral genome with the small dots of N protein, which, however, are difficult to visualize in this way due to the low level of fluorescent probe in such structures, as seen with PUUV-N (Fig 5A, arrows). This is not the case for the big granules observed with TULV, which are not stained by viral probes.

Altogether these results indicated that the N protein in association with genomic RNA could constitute some viral structures partly interacting with the secretory pathway, where the glycoproteins assemble, and with the endocytic pathway, suggesting that viral particles could be secreted not only at the level of the Golgi but also via other pathways.

#### Different nucleocapsid protein organizations

**Fig 6:**
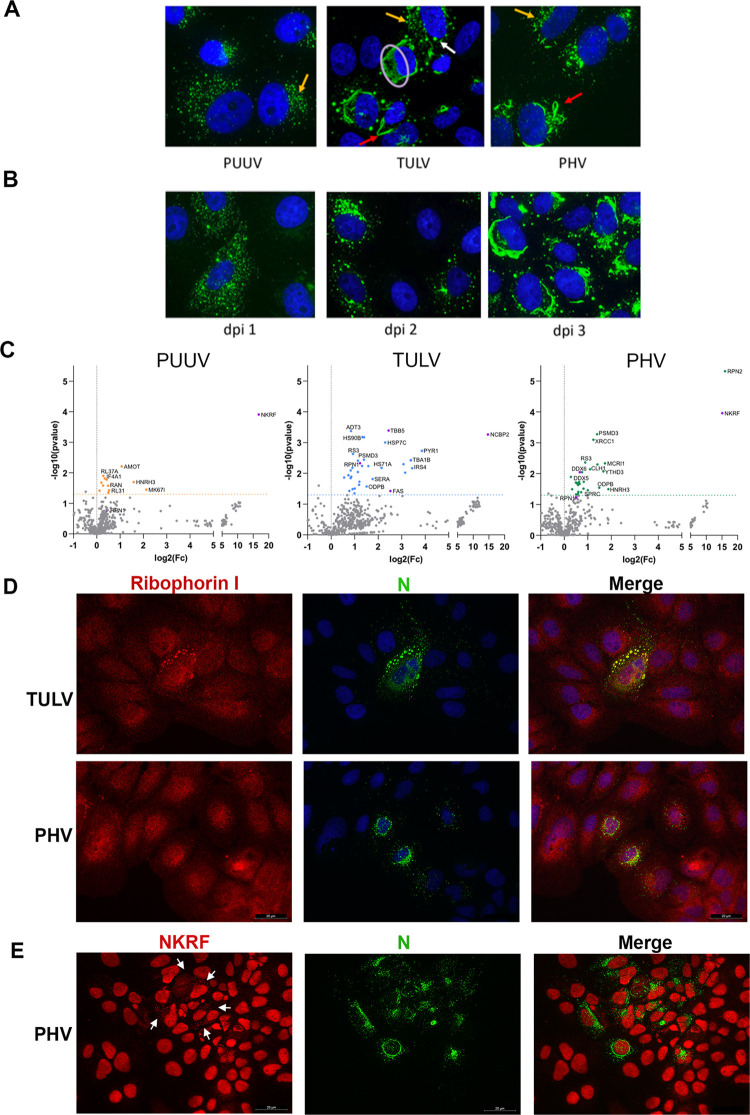
Interaction of the nucleocapsid of PUUV, TULV and PHV with cellular proteins. Panel (A) shows the different organizations of PUUV-N, TULV-N or PHV-N, identified by intracellular detection at dpi 3 in infected Vero E6 cells (green staining). Small dots of N protein are marked with yellow arrows, filaments with red arrows, bigger granules with white arrows and aggregates are circled in pale purple. Nuclei are counterstained with DAPI (blue staining). In (B), kinetics of TULV-N distribution is shown at dpi 1, 2 and 3. In (C), interactors of the N of PUUV, TULV and PHV identified by mass spectrometry analysis are shown. Cellular partners, statistically significant (p-value<0.05), are colored in orange, blue and green for PUUV, TULV and PHV, respectively. Further explored interactors are colored in violet. In (D), HuH7 cells were infected with TULV or PHV at a MOI of 0.5 and stained for ribophorin I (red) and viral N (green). Co-localization appears in yellow in the merge panel. In (E), HuH7 infected with PHV were stained for NKRF (red) and viral N (green). White arrows highlight the distribution of small dots of NKRF in the cytoplasm of infected cells while NKRF mostly localized in the nucleus of non-infected cells.

As expected, the viral N proteins were found to localize in the cytoplasm of infected cells (Fig 1B). However, different organizations of the protein were seen, depending on the virus and the kinetics of infection (Fig 6A and 6B). In PUUV-infected cells, N protein was found as small dots (yellow arrows), also predominating in cells infected by TULV or PHV (Fig 6A). Nevertheless, the nucleocapsid organization of these two viruses was more heterogeneous and included N filaments (red arrows). In TULV infected cells, the N protein also formed amorphous aggregates in the cytoplasm (purple circle) as well as big granules (white arrow). We explored the way these organizations appear by performing a kinetics study using TULV-infected cells, which showed the highest variability in N protein morphologies. At dpi 1, only small dots were present in the cytosol of TULV-infected cells, similarly to what was observed for PUUV-N infected cells. The other structures appeared later from dpi 2: first filaments, then granules of bigger size and then, at dpi 3, amorphous aggregates began to fill a larger area in the cytosol (Fig 6B).

#### Cellular partners of the N proteins

The different outcomes of PUUV, TULV and PHV infection, together with the different organization and association of N proteins with cell compartments, led us to search for cellular partners in complex with this viral protein by mass spectrometry analysis. N proteins of PUUV, TULV and PHV, fused to a strep-tag were pulled-down from transfected human embryonic kidney, HEK293T cells, and significant interactions of cellular proteins with the viral nucleocapsids were quantitatively determined. Overall, more than 500 hits were identified for each N protein. However, when considering the statistically significant hits with more than one unique peptide, and additionally filtered for p<0.05, and FC>2, the list of identified proteins shortened (Tables 2–4).

**Table 2:** Human proteins found in complex with PUUV-N in HEK293T cells.

Gene Name^1^	fold change^2^ PUUV vs NI	Anova. P<0.05	Function
NKRF (2)	1.1e+5	0.00012	NFκB repressing, silencing of IFNβ & cytokine promoters
MK167 (4)	4.36	0.03569	FHA interacting nucleolar protein, cell cycle progression
HNRH3 (7)	2.99	0.02015	RBP, heterogenous nuclear RNP H3
AMOT (4)	2.10	0.00615	Angiomotin, tight junction maintaining

**Table 3:** Human proteins found in complex with TULV-N in HEK293T cells.

Gene Name^1^	fold change^2^ TULV vs NI	Anova. P<0.05	Function
NCBP2 (1)	2.6e+4	0.00054	Nuclear cap-binding protein subunit 2
PYR1 (6)	14.5	0.00184	CAD protein, nucleotid synthesis, cell proliferation
IRS4 (6)	10.9	0.00679	Insulin receptor substrate: signaling pathway regulator
TBA1B/1C (1)	10.4	0.00375	Tubulin α, microtubule: intracellular transport
RUVB1 (4)	8.87	0.00953	DNA helicase, transcriptional activation
TBA1A (1)	8.44	0.00503	Tubulin α, microtubule: intracellular transport
FAS (4)	5.72	0.03757	Fatty acid synthase: palmitate synthesis, IIR, apoptosis
TBB5 (5)	5.44	0.00040	Tubulin β, microtubule: intracellular transport
HSP7C (24)	4.92	0.00099	Heat shock protein 70 kD: protein folding
HS71A/1B (13)	4.41	0.00668	Heat shock protein 70 kD: protein folding
SERA (2)	3.40	0.01531	D-3-phosphoglycerate dehydrogenase, amino acid synthesis
CLH1 (6)	2.99	0.00578	Clathrin heavy chain, endocytosis
ODPB (1)	2.84	0.00267	Pyruvate dehydrogenase mitochondrial metabolism
TBB2B (1)	2.63	0.000675	Tubulin β, microtubule: intracellular transport
PSMD3 (5)	2.62	0.003597	Proteasome 26S subunit: protein degradation
HS90B (4)	2.51	0.000665	Heat shock protein 90 kD, folding, maturation
ATD3A (5)	2.48	0.005770	ATPase, mitochondrial transport, apoptosis regulation, antiviral
RPN1 (2)	2.34	0.004701	Ribophorin1, glycosyltransferase: N glycosylation of protein
TCPG (4)	2.30	0.018762	Molecular chaperone, TCP1 complex: folding
RS2 (9)	2.28	0.023629	40S ribosomal protein S2, mRNA translation
NDUA4 (1)	2.20	0.003888	Subunit of mitochondrial respiratory chain complex
RUVB2 (6)	2.19	0.009539	DNA helicase, transcriptional regulator
TBB4B (3)	2.00	0.031129	Tubulin β, microtubule: intracellular transport

**Table 4:** Human proteins found in complex with PHV-N in HEK293T cells.

Gene Name^1^	Fold change^2^ PHV vs NI	Anova. P<0.05	Function
RPN2 (2)	6.4e+4	4.8e-6	Ribophorin II, glycosyltransferase: N glycosylation of protein
NKRF (2)	3.5e+4	0.00019	NFκB repressing, silencing of IFNβ & cytokine promoters
HNRH3 (7)	3.7	0.03189	RBP, heterogenous nuclear RNP H3
MCRI1 (3)	3.3	0.00466	MAPK-regulated corepressor-interacting protein 1
YTHD3 (3)	3.2	0.00848	RNA binding protein: mRNA processing and stability
ODPB (1)	2.81	0.02863	Pyruvate dehydrogenase mitochondrial metabolism
RBM33 (1)	2.66	0.00504	RNA binding protein
PSMD3 (5)	2.63	0.00053	Proteasome 26S subunit: protein degradation
XRCC1 (1)	2.36	0.00079	DNA repair transcription coupled
CLH1 (6)	2.14	0.00723	Clathrin heavy chain, endocytosis
SPRC (2)	2.00	0.03463	Secreted protein, interact with matrix and cell shape

^1^ the number of unique peptides is indicated in brackets.

^2^ proteins with a fold change >2 are considered and when found associated with PUUV-N and PHV-N they are highlighted in blue and when associated with TULV-N and PHV-N, in beige.

Only four proteins were found in complex with PUUV-N (Table 2), 23 with TULV-N (Table 3), and 11 with PHV-N (Table 4). These proteins are implicated in a broad range of functions, such as the innate immune response, protein folding and stress response. Cellular factors involved in protein N-glycosylation (RPN1 and RPN2) or P-bodies’ assembly such as different helicases were also identified. Cellular partners of PUUV-N were mainly involved in RNA processing. Partners associated to TULV-N were implicated in apoptosis (fatty acid synthase, FAS), cytoskeleton organization (tubulin subunits) and protein quality control (heat shock proteins, proteasome), while PHV cellular partners were mostly implicated in cell signaling and RNA processing. Of interest, the repressor of NFκB (NKRF), which is involved in silencing of IFNβ [62], was found with a high score associated with both PUUV-N and PHV-N, while NCBP2, a cap binding protein, was identified with a high score with TULV-N.

**Fig 7:**
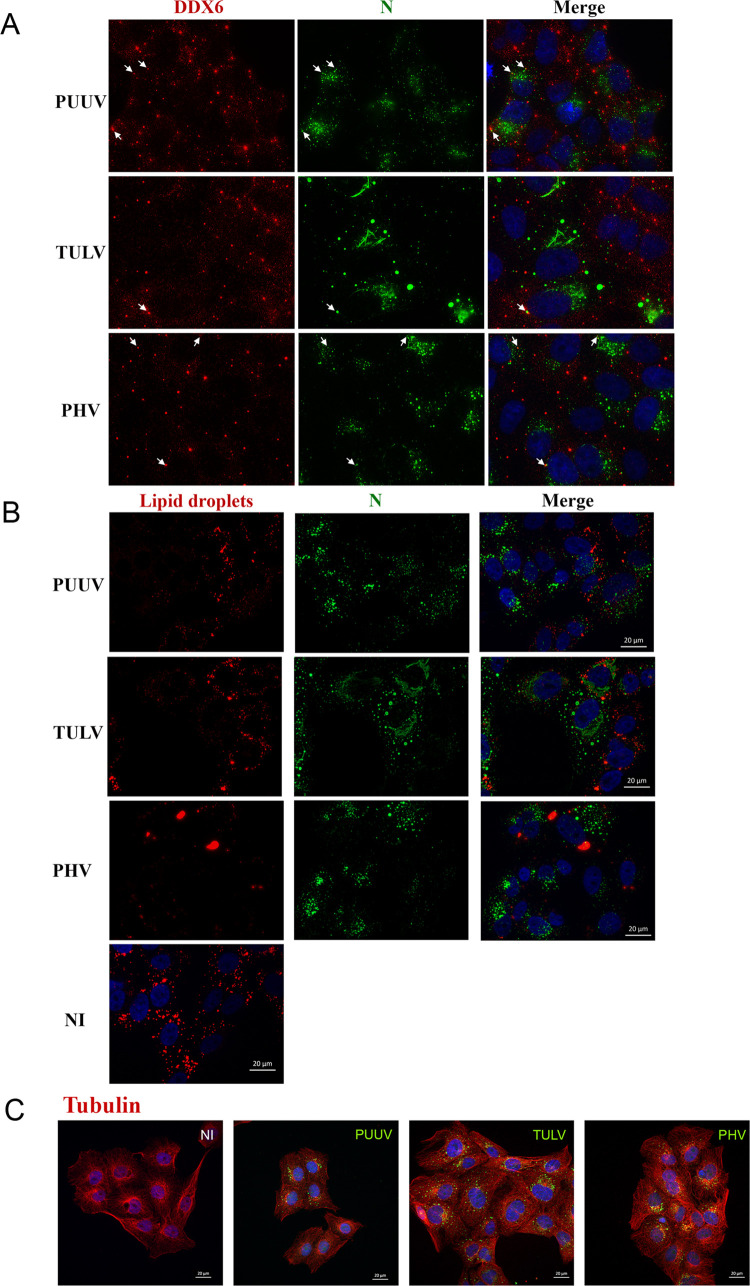
Immunostaining validation of identified interactions of N protein with human proteins. Immunofluorescence co-staining of infected Vero E6 cells was performed as in Fig 4. The localization of viral N protein (green) with markers of P bodies, DDX6, and lipid droplets is shown in (A) and (B) respectively. In (B), the distribution of lipid droplets in non-infected cells (NI) is shown for comparison with infected cells. The co-staining of N and tubulin is shown in (C). Cellular markers are labelled in red while nuclei are counterstained in blue (DAPI).

The cellular distribution of some of the identified proteins, in complex with viral N proteins, was tested by immunofluorescence co-staining in infected Vero E6 cells or HuH7 cells. As shown in Fig 6D, the big granules of TULV-N were found to co-localize with human ribophorin I in infected HuH7 cells. Concerning NKRF and NCBP2, we could not see co-localization with viral N proteins (Figs 6E and S7). This could be explained by their major nuclear localization, as it is often the case of signaling proteins shuttling between nucleus and cytoplasm. However, in HuH7 cells infected with PHV we observed a discrete distribution of small dots of NKRF in the cytoplasm of infected cells (Fig 6E, white arrows), not visible in non-infected cells or cells infected with TULV or PUUV (S7 Fig). As shown in the volcano plot where the MS data were filtered only according to a p value <0.05, PHV-N interacted with the RNA helicase, DDX6, one of the major constituents of P-bodies and a few dots were co-stained for PHV-N and DDX6 (arrows, Fig 7A) in infected Vero E6 cells. This was also the case for very few dots of PUUV-N and TULV-N, which was also consistent with N associating with other RNA binding proteins and proteins involved in transcriptional regulation. Since TULV-N was interacting with FAS, a multi-enzyme protein responsible for the production of palmitate from acetyl-CoA and malonyl-CoA, but also described for its role in innate immune response and apoptosis, we performed lipid droplets labeling of infected cells with fluorescent BODIPY a metabolite incorporating such structures. A large number of bright lipid droplets were visible in non-infected (NI) Vero E6 cells. We observed that this staining was reduced when cells were infected with TULV, but also with PUUV, suggesting an impact of orthohantavirus infection on lipid metabolism. In the case of PHV, beside reduce detection of lipid droplets in infected cells, a coalescence of lipid droplets was also observed (Fig 7B). While we did not observe important co-localization of TULV-N and PHV-N with tubulin filaments, fluorescence intensity of tubulin staining increased in such infected cells (Figs 7C and S4B) as compared to PUUV-infected cells or to the non-infected control, corroborating the high number of peptides from different tubulin subunits found in association with TULV-N. This suggests a reorganization of the microtubule network by the infection as also shown by staining of actin and vimentin filaments of the cytoskeleton (S4B and S4D Fig, respectively).

### Interferon response is differentially affected by orthohantavirus infection in human and bank vole cells

**Fig 8:**
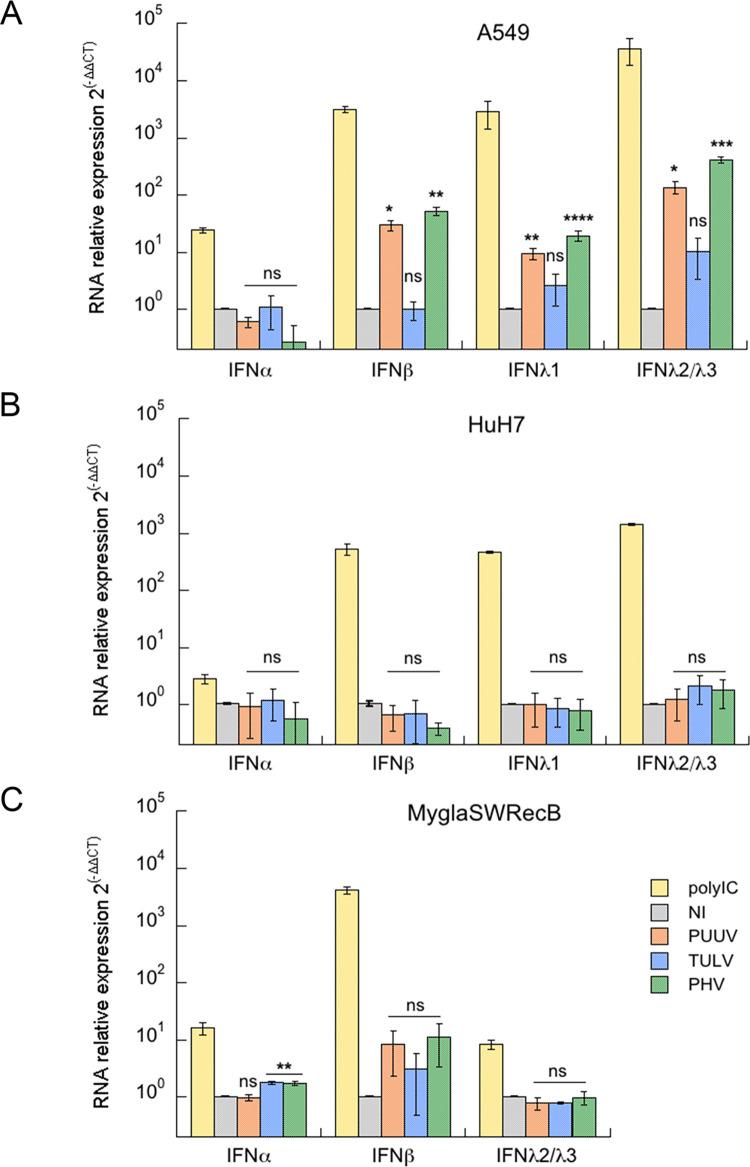
Regulation of IFN expression in infected human and bank vole cells. Human A549 (A) and HuH7 (B) cells and rodent MyglaSWRecB cells (C) were left non-infected (grey bars) or were infected with PUUV (orange bars), TULV (blue bars) or PHV (green bars) at a MOI of 0.5. As positive control, a condition where cells were treated with poly-IC was included (yellow bars). For each condition, the mRNA expression of the different IFN type I (IFNα and β) and type III (IFNλ1, λ2 and λ3), was compared with non-treated cells (2^-ΔΔCT^) by RT-qPCR. Error bars correspond to the standard deviation from the mean determined from at least three replicates of three independent samples. Statistical analysis showed non-significant (ns) and significant variations relative to non-infected cells with p-values p<0.0332 (*), p<0.0021(**), p<0.0002 (***) and p<0.0001(****).

Because viruses elicit antiviral responses that they must counteract in order to propagate, we wondered whether differential regulation of interferon might contribute to the observed differences in the life cycle of orthohantaviruses. Therefore, we quantified the production of IFNα, IFNβ and IFNλ (λ1 and λ2/λ3) mRNA by performing RT-qPCR in human (HuH7 and A549) and bank vole (MyglaSWRecB) cell lines infected with PUUV, TULV or PHV. The experiment was conducted at dpi 5 (Fig 8), which corresponds to the peak of innate immune response as shown in our previous study exploring the regulation of genes expressed in A549 human cells infected with PUUV [30]. We included this cell line in the present study for comparison. Poly-IC was used to control the level of IFN activities in human and bank vole cell lines. Although IFNα was poorly activated, the two other IFN families, IFNβ and IFNλ, were activated in human A549 cells treated with poly-IC, showing around 10^3^-fold change in relative mRNA expression levels as compared to untreated cells. However, none of the three viruses induced IFNα, whereas PUUV and PHV, but not TULV, significantly activated IFNβ, IFNλ1 and IFNλ2/λ3 (Fig 8A). In contrast, none of these orthohantaviruses induced a significant amount of IFNs in HuH7 cells, despite the fact that activation of IFNβ, IFNλ1 and IFNλ2/λ3 occurred when poly-IC was used (Fig 8B).

Little is known about bank vole transcriptome and proteome, but interestingly, a bank vole transcriptome assembly has recently been produced by a high throughput sequencing experiment [44]. We used it to search for IFNα, IFNβ and IFNλ2/λ3 mouse orthologs to design primers for amplification of these genes in the *M*. *glareolus* cell line, MyglaSWRecB. It is noteworthy that no equivalent of human λ1 (IL29) can be found in house mouse genomes. Poly-IC induced high levels of IFNβ mRNA (>10^3^-fold change) in this cell line, whereas significant but lower levels of IFNα and IFNλ2/λ3 were activated. Therefore, the very low fold change (5 times) in IFNβ RNA expression found in MyglaSWRecB cells infected with the three orthohantaviruses (Fig 8C), could be compared to the low activation level in A549 cells. However, statistical analysis revealed that this low level of IFNβ appeared to be not significantly different from the IFNβ level in non-infected cells. This suggests that PUUV and PHV, replicating in MyglaSWRecB cells, do not induce appreciable levels of type I and type III IFNs at dpi 5, whether they release infectious particles (PUUV) or not (PHV). This is also the case of TULV, even though it does not replicate efficiently in this cell line.

### Differential regulation of gene expression in bank vole cells infected with PUUV or PHV is mainly related to the innate immune response

**Fig 9:**
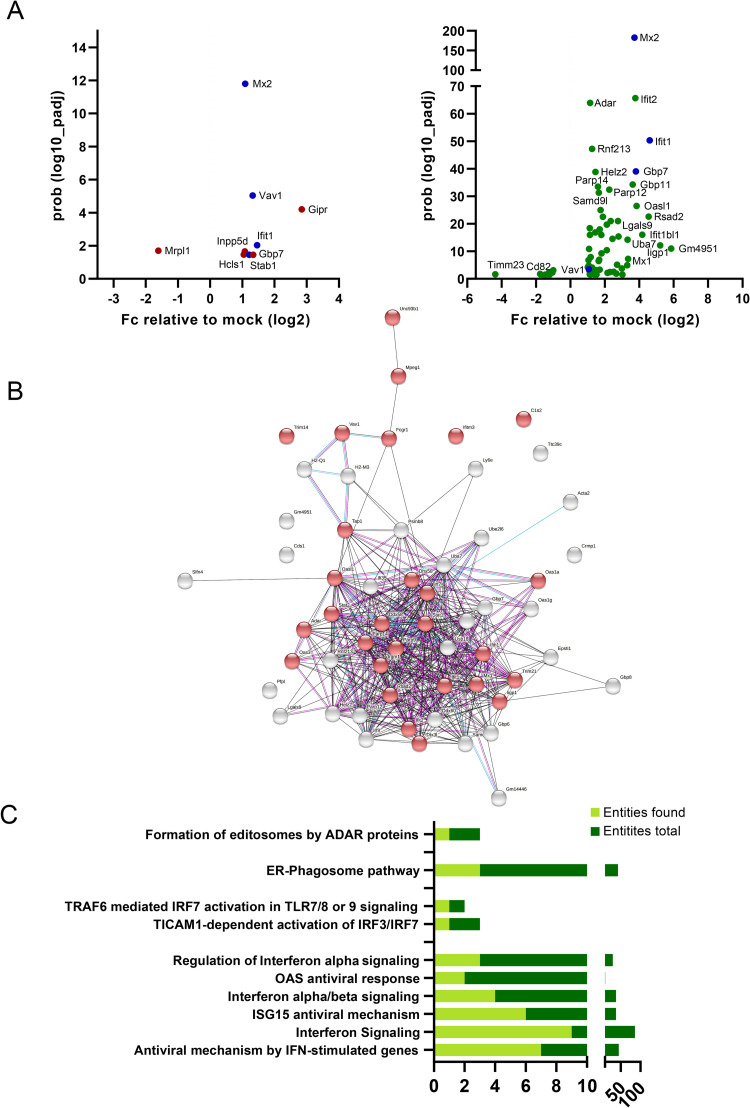
Transcriptome analysis of MyglaSWRecB bank vole cells infected with PUUV and PHV. Graphs in (A) represents the upregulated genes in MyglaSWRecB cells infected with PUUV (left panel) and PHV (right panel), as compared to non-infected (NI) cells, which were identified by RNA-Seq using the *Mus musculus* reference genome, GRCm38/mm10. Graphs were generated using GraphPad Prism software by plotting the -log10 of the adjusted p-value against the log2 of the fold change in gene expression. Genes colored in blue were up regulated under both viral conditions, while PUUV- and PHV-specifically induced genes are shown in orange and green, respectively. In (B) *M*. *musculus* transcriptome data for PHV-infected bank vole cells were analyzed using the on line-software STRING. The up-regulated genes are represented as an interaction network, with the nodes involved in the immune response colored in red, according to the GO terminology. In (C), the most significantly enriched pathways, compared to non-infected cells, are listed based on Reactome classification. The number of genes identified in RNA samples of PHV-infected cells (entities found, light green), is shown relative to the total amount of genes involved in the specific corresponding functions that are indicated (total entities, dark green).

**Fig 10:**
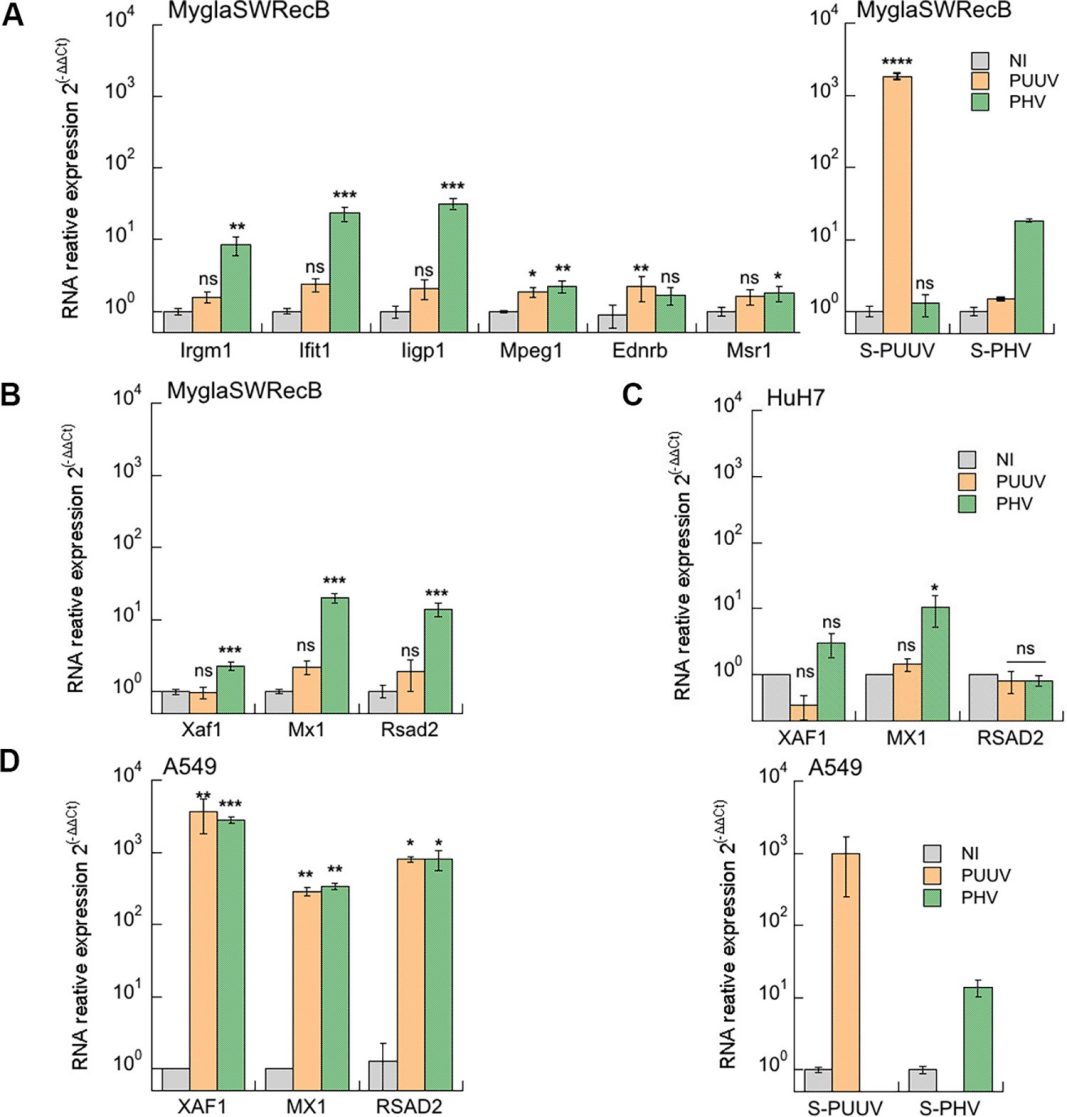
Quantitative RT-qPCR confirmation of gene expression in bank vole cells. In (A), relative differential expression of Irgm1, Ifit1, Iigp1, Mpeg1, Ednrb, Msr1 genes measured by RNA-Seq was validated by RT-qPCR using RNA extracted at dpi5 from MyglaSWRecB that were either not infected (NI, grey bars), or infected with PUUV (orange bars) or PHV (green bars). In (B), the expression of Xaf1, Mx1 and Rsad2, was examined in MyglaSWRecB cells and, the expression in infected HuH7 and A549 cells of their human counterparts RSAD2, XAF1, and MX1 is shown in (C) and (D) respectively. The expression of RNA corresponding to the S segments of PUUV and PHV is shown in the right panels of (A) for MyglaSWRecB and of (D) for A549-infected cells. Error bars correspond to the standard deviation from the mean of samples tested in triplicate and statistical analysis performed as shown in Fig 8.

Beside a weak IFNβ activation in infected human A549 cells and infected bank vole MyglaSWRecB cells, we observed a discrepancy in the IFNλ2/λ3 expression in these two cellular models (Fig 8C versus 8A). We previously described a transcriptome analysis of A549 infected by PUUV [30]. In order to broaden the comparison of cellular regulation induced by orthohantaviruses to the situation in which they are adapted to the bank vole (PUUV) or not (PHV), we then performed quantitative transcriptomic analysis by sequencing RNA extracted from MyglaSWRecB cells infected with one or the other of these two viruses. A significant proportion of the RNA-Seq data (28%) could be mapped to the reference genome of the house mouse. As expected, the viral genes specific of each virus were identified in the corresponding infected cells and not in the uninfected control cells. The up- and down-regulated genes are listed in the Tables A and B in S1 Table and the results visualized by volcano plots (Fig 9A). Only a few genes were found to be differentially expressed in PUUV infected bank vole cells (8 up-regulated and 1 down-regulated) compared with a larger number of genes being modulated by PHV infection (58 up-regulated and 9 down-regulated). Twenty-five of these 58 DEG were also found to be significantly upregulated (Table D in S1 Table) when the reads (82% of the reads in total) were mapped to the recently assembled and annotated draft bank vole genome [50]. Interestingly, most of the genes activated in bank vole cells by PHV, but not by PUUV, belong to the interferon-signaling pathway (Fig 9B) and were also found as being upregulated in our previous study, in human A549 cells infected with PUUV [30]. This allowed us to select DEG from both the annotated mouse and bank vole genomes, involved in different pathways such as, IFN-dependent antiviral response (Mx1, Ifit1, Iigp1, Rsad2, Ednrb), apoptosis (Irgm1, Xaf1), or lipid regulation (Msr1), which could be correlated with observations of proteomics or viral cycle restrictions obtained in the present study. To assess the reliability and reproducibility of the results obtained in the RNA-Seq analyses, we validated expression of these selected genes by RT-qPCR, in independent samples of MyglaSWRecB cells infected with PUUV or PHV (Fig 10A). The observation of a discrepancy between the degree of upregulation of genes by PHV as compared to PUUV in the transcriptomic analysis was confirmed for several genes such as Ifit1, Irgm1, and Iigp1 for which statistical analysis found a significant activation only by PHV. Interestingly only Mpeg1 and Ednrb appeared to be significantly activated by PUUV although the level of expression was quite low. Expression of three genes, known to be IFN-induced (Mx1, Xaf1 and Rsad2), were compared in bank vole and human cells. Of particular interest, the upregulation of Rsad2 in bank vole cells was induced by PHV but not by PUUV (Fig 10B). A similar profile was seen for MX1 in infected-HuH7 cells, while RSAD2 and XAF1 were not activated (Fig 10C). In contrast, RSAD2, along with MX1 and XAF1, was induced at high levels by both viruses in human A549 cells (Fig 10D). The presence of the viral RNA segments, which was validated by the RNA-Seq analysis, was also evidenced by RT-qPCR of an S segment sequence in the RNA samples from infected bank vole and human cells (right panels of Fig 10A and 10D respectively).

These results showed that production of infectious PUUV particles in bank vole cells is associated with low expression levels of IFN-induced genes. In contrast, the PHV-induced higher upregulation of genes involved in the antiviral response corroborates the observed restriction of infectious particle release.

## Discussion

Orthohantaviruses, hosted by various species of rodents, can also infect humans upon occasional transmission. Due to the difficulties in defining an appropriate animal model for orthohantavirus pathology and to ethical and management limitations associated with *in vivo* studies, it is of the uttermost importance to define pertinent cellular models to obtain information on the relationship of these viruses with their hosts [63,64]. A number of publications have previously described the susceptibility to infection of various cell lines, from either human [65,66] or rodent [13,39] origin. However, due to the use of different experimental conditions, a clear picture of which cells are best suited to address biological issues of orthohantavirus infection is yet missing. Our study not only reflects some host specificity of PUUV, TULV and PHV, but also shows differences in cell restriction for production of infectious hantaviral particles and regulation of cellular factors depending on the virus species and host cell types.

### Different levels of cell restriction in the production of infectious hantaviral particles

We observed different patterns of infection by PUUV, TULV and PHV in human and rodent cell lines. HuH7 derived from human hepatocytes were susceptible to infection by the three orthohantaviruses, as were differentiated THP1 cells, which could be useful for *in vitro* studies of viral propagation since mononuclear phagocytic cells are involved in pathogenicity [3]. Also, even though hepatocytes are not the main targets of orthohantavirus infection, hepatic functions can be altered [38]. Although it is widely reported that Old-World pathogenic orthohantaviruses, such as PUUV, damage primarily glomerular and tubular functions of the kidneys, the targeted cellular type in the organ is not fully defined. It has been shown that primary renal cells are weakly susceptible to hantaviral infection [65]. We did not have permissive human kidney cells in our study but it would be of interest to test different renal human cell lines for their susceptibility to infection. In other cases where human cells could be infected, preferential infection by PUUV (A549) or by TULV (Caco2) occurred. Since it has been shown that Vero E6 cells secrete IFN-λ in response to orthohantavirus infection [42], this cytokine could participate to some of the observed differences. However, in this study by Prescott et al., IFN-λ appears late in infected Vero E6 cells (not before dpi 12), whereas viral stocks were prepared at dpi 7, therefore minimizing a potential impact in infectivity. In addition, we showed that neutralization of IFN-λ by treatment with anti-IL29 or -IL28A antibodies did not improve infectivity of PUUV, TULV or PHV in Vero E6 cells and in human HuH7 cells.

In cell lines derived from rodent hosts, the scenario was different, and species-specificity of infection was observed. PUUV was shown to be infectious towards cells derived from the kidney or the respiratory tract of *M*. *glareolus*, while conversely TULV, which is harbored by another vole, *M*. *arvalis*, and replicated at high titer in Vero E6 cells was not able to significantly infect cells derived from this heterologous bank vole reservoir. These findings prove the bank vole cell models used in our study to be a valuable tool to study orthohantavirus biology.

The relationship between each of the three orthohantaviruses with the different human and bank vole host cells was investigated by assessing production of infectious particles and by quantifying the amount of viral RNA in cellular lysates and supernatants. In the case of the human HuH7 cells, the infectivity of the supernatant was much reduced in cells infected with TULV or PHV and not even measurable when infected with PUUV. Their reduced infectivity measured in secondary infection correlated with the low amount of genomic RNA detected in the HuH7 supernatant, confirming that few virions were released and suggesting this human cell line is not optimal to achieve complete hantaviral cycle. Interestingly, in the case of the rodent cell lines, we found that production of PUUV infectious particles was as efficient in MyglaSWRecB as in Vero E6 cells. Production of PUUV stocks in this cell line should be considered for future studies. In contrast, infection of MyglaSWRecB by TULV appeared to be restricted at an early step of the viral cycle, and replication could not be detected in these cells, explaining the absence of infectious particles. This, in addition to the fact that TULV, but not PUUV, has been shown to infect cells derived from its *M*. *arvalis* natural host [67], was in line with the host-specificity of orthohantaviruses. A third scenario applied to PHV, since, although the bank vole cells were susceptible to infection by this virus, no infectious particle was detected in their supernatant, suggesting that cellular restrictions operated later in the viral cycle, as compared to TULV, which was blocked at an early step.

Despite difficulties to find mature PUUV particles in Vero E6, HuH7 and MyglaSWRecB infected cells by EM analysis, due to its low viral titers, clusters of mature viral particles appeared in polarized regions of TULV-infected Vero E6 cells as membrane protrusions in area of virus release (S5 Fig). In contrast, no virion was seen at the surface of infected HuH7 cells. Surprisingly, presence of unexpected tubular organizations was visible at the surface and the extracellular space of both Vero E6 and HuH7 cells infected with TULV, indicating that virus particles may bud at the plasma membrane of Vero E6, as shown for New World orthohantaviruses, i.e. Black Creek Canal virus and SNV [20,68]. We hypothesized that the tubular structures could be constituted of viral glycoproteins present at the surface of the cells, as supported by the strong Gn fluorescent staining observed at the membrane of TULV-infected Vero E6 cells. Accumulation of the viral glycoproteins inserted into lipid layers at the cell surface, could then generate extruding empty tubes formed of glycoproteins. However, their empty morphology seemed to exclude that they were mature viral particles and therefore, infectious, since they were also found in HuH7 cells in absence of infectivity. This finding and the absence of infectious particles in TULV-infected HuH7 cells also raises the question of a possible cell-to-cell passage of viral proteins and/or particles in infected HuH7 cells.

Because of its low titer, we do not know whether PUUV-infected cells produce such tubular structures. Of note, under conditions for which a similar infection was detected by intracellular Gn staining, we did not observe expression of Gn at the plasma membrane of PUUV-infected cells, in contrast to TULV-infected cells. The role of virus-induced tubes is currently unknown. They might result from overexpression and accumulation of viral glycoproteins at the surface of cells as described in the case of SNV-infected Vero E6 cells [68] with no impact on the production of infectious particles, or they could represent “interfering particles”, decreasing infectivity of the supernatant. Nevertheless, in the case of Vero E6 cells infected by TULV, it seems that these structures could be released together with the complete virions, forming “clusters” of infectious particles. It has been previously shown that collective infectious units of vesicular stomatitis virus enhance its efficiency of infection in a cell-type dependent manner [69]. A similar mechanism could be hypothesized for orthohantaviruses, which would then account for the higher titers observed in TULV stocks. It is to be noticed that cryo-EM analysis of purified New World orthohantaviruses, which bud at the plasma membrane of infected cells, exhibited heterogeneous morphologies from round to tubular as well as mixed irregular particles [70]. Our hypothesis is also supported by a recent study in which transmission EM has been used to visualize viral particles at the interspace between cells infected by Old World orthohantaviruses [70,71].

We therefore hypothesized that the restriction in the secretion of infectious particles of TULV in HuH7 cells and of PHV in bank vole cells is not similar and is not due to cytokines associated to the production of mature particles. The absence of released viruses could be due to a defect in particle maturation and to degradation of viral particles, respectively.

### Interaction of orthohantaviruses with cellular factors

Another approach, used to investigate how viral maturation and life cycle proceed, was to search for interactions of viral proteins with cell compartments and/or factors that could be diverted by viruses [72]. In this respect, considering their multiple functions in viral replication, viral assembly and interaction with the innate immunity [73], we were interested in the various cellular organizations of the different orthohantaviral N proteins, appearing as dots in cells infected by all three orthohantaviruses, but also as N filaments with PHV and TULV, and as big granules and amorphous aggregates specific of TULV-N. It is known that viral particles of other bunyaviruses assemble at the level of the Golgi, so the partial localization of the N protein with different compartments involved in secretion was not surprising. On the other hand, a partial localization with endocytic vesicles, more particularly the recycling endosomes, is of interest, since such interaction has been demonstrated for New World orthohantaviruses, as to allow efficient release of ANDV particles budding at the plasma membrane [61]. However, as expected from a multifunctional protein, we never observed complete co-localization of N protein with markers of cell compartments, although their cellular organization was perturbed, in particular in TULV-infected cells. Data from the literature report various N protein localizations in cell lines of different origin infected by different orthohantaviruses. It will then be of interest to further investigate other cell compartments involved in cell stress, degradation or other functions, such as mitochondria, stress granules or autophagic vacuoles, since some orthohantaviruses have been described to interact with such subcellular compartments [27,74–76]. However, *in situ* hybridization demonstrated that the N-filament organizations of TULV and PHV were associated with both genomic and antigenomic RNA and could constitute viral replication sites in infected Vero E6 and HuH7 cells. The different forms adopted by the N protein in TULV-infected cells could relate to its replication rate, illustrated by a high genome copy number (25 times higher than PUUV and 7.5 times higher than PHV), correlating with a high amount of N protein. As previously observed with the non-structural protein, NSs, of TULV [30], intrinsic properties in the nucleotide or amino-acid sequences, could structurally favor the efficient synthesis of N proteins or its stability due to its structural organization. In this regard, the co-localization of the big granules of TULV-N with ribophorin I, a sub-unit of the oligosaccharide glycosyltransferase located in sub-compartments of the ER, is of interest. Therefore, TULV-N granules and aggregates accumulating during infection, excluding cell compartment markers, may represent virus-induced sites of storage, protecting cells from excessive stress induced by an excess of viral proteins. TULV-N aggregates could also prevent the access of probes or antibodies, explaining why we were not able to visualize interaction with cellular or viral components associated to these peculiar N protein structures. These results, along with mass spectrometry data looking for partners of N proteins, strengthen the implication of the N protein in a variety of cellular functions, which could be diverted by orthohantaviruses to their own advantage. Our findings and confirmation by immunofluorescence co-staining of interaction of N protein with tubulin subunits, support the importance of microtubule components for cytoskeleton integrity and the production of infectious orthohantavirus particles [22]. Also, the interaction of N protein with DDX6 a marker of P-bodies is comforted by results from the literature showing that SNV N protein co-localize with P-bodies suspected to be important for cap snatching [23] and has been recently shown to occur in cells transfected with PUUV-N encoding plasmid [77]. P-bodies are cytoplasmic structures, which are implicated in cellular mRNA turnover. In line with this, TULV-N, which is also associated to viral RNA, was found in complex with NCBP2, a protein involved in cap snatching. Consistently, we also observed interaction of N with proteins involved in RNA binding, transcription and translation. Finally, the interaction of the nucleocapsids with Fatty acid synthase (FAS) and related factors corroborated our immunofluorescence data indicating that the distribution and morphology of lipid droplets were altered in infected cells. Fatty acid metabolism is thought to have a central role in the regulation of the innate immunity and apoptosis [78,79]. We suspect that viral infection may be related to cellular physiology of fatty acids. The importance of lipid metabolism and in particular FAS for viral maturation has been demonstrated for flaviviruses and some respiratory viruses. It would be of interest to further investigate the effect of inhibitors of fatty acid synthesis on the production of infectious orthohantaviruses [80,81].

### Differential regulation of gene expression in rodent and human cell lines depends on orthohantavirus species

**Fig 11:**
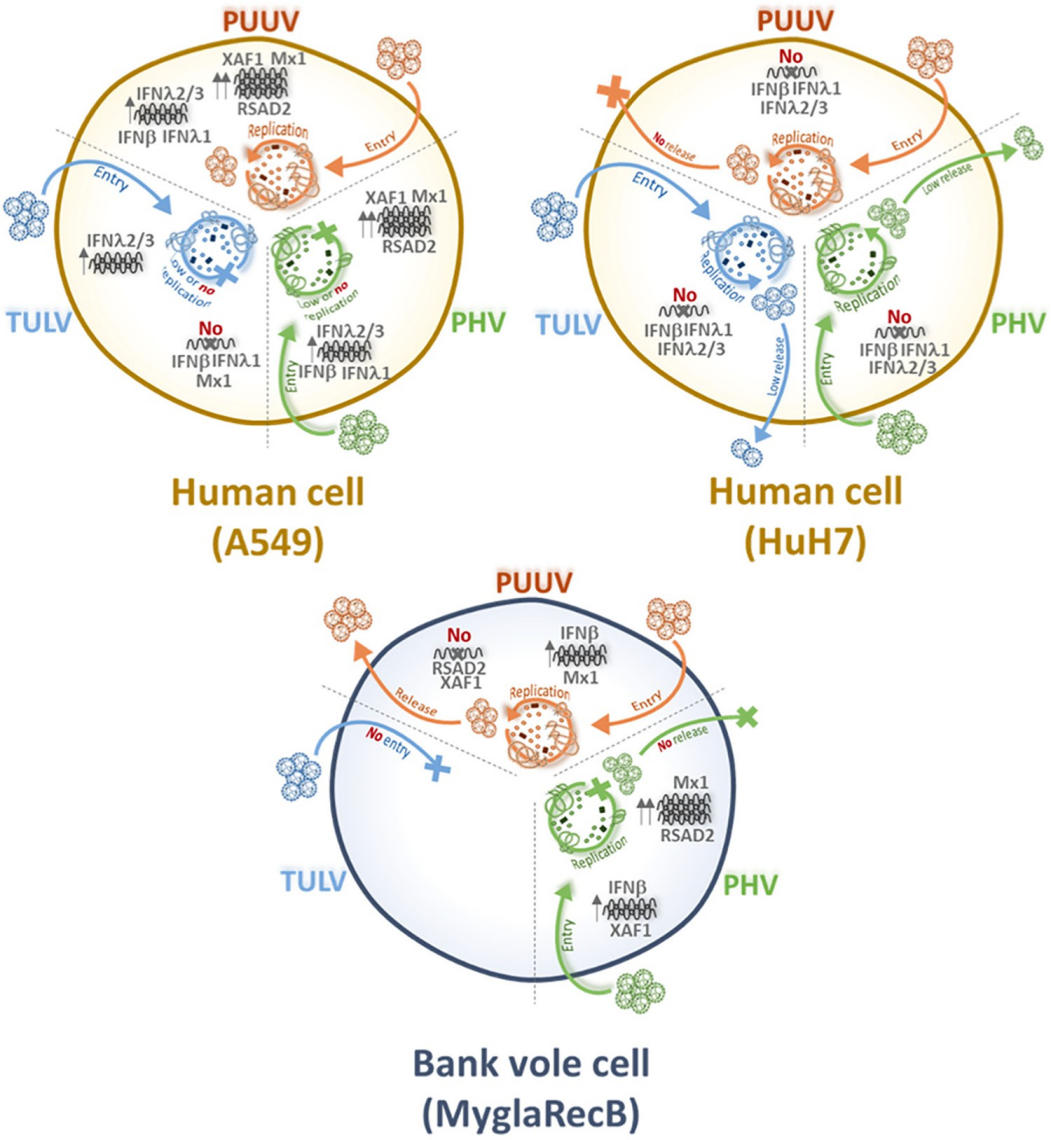
Schematic representation of the different interactions of orthohantaviruses with human and rodent cells. The cell susceptibility to PUUV- (orange), TULV- (blue) and PHV- (green) infection, viral replication and production of infectious particles in human A549 and HuH7 cells and bank vole MyglaSWRecB cells, are schematically summarized. Colored arrows, as indicated for each of the three viruses, indicate the main steps of the viral cycle i.e. entry, replication and release. Levels of expression of IFNs and ISGs are visualized as black RNA strands and their relative amount by grey arrows next to their name.

It is known that secretion of anti-viral cytokines, such as IFNs, released from infected cells, blocks the propagation of viruses. The different capacities of orthohantaviruses to antagonize anti-viral activities may be related to disparities in the production of infectious orthohantaviruses by human or rodent cells as schematically summarized in Fig 11. We have previously described differences in IFN production by human A549 cells infected with PUUV or TULV [30] and demonstrated that induction of IFN and IFN-regulated genes occurs late during infection. This was confirmed in the present study, and the addition of HuH7 and MyglaSWRecB cells made it clear that expression of mRNA encoding IFN was not detectable in HuH7 cells, in which few viral particles were produced, and was not significantly detectable in rodent cells, whether they produced infectious particles (PUUV) or not (TULV and PHV). This supports an involvement of other regulatory functions than the early IFN induction.

It is important to understand how orthohantaviruses persist in rodent cells and how the fine specificity to rodent hosts is achieved. In this context, Spengler and colleagues, have successfully infected the deer mouse (*Peromyscus maniculatus*), the natural reservoir of SNV, with this orthohantavirus. On the other hand, the authors have demonstrated that ANDV, which is hosted by another rodent (*Oligoryzomys longicaudatus*), is cleared about 50 days post infection when experimentally inoculated to deer mice [82]. Comparison of PUUV and PHV, both of which infected MyglaSWRecB cells, whereas only PUUV produced infectious particles, offer a good model to address these questions. At present, we have limited knowledge of the genetics of the many different rodent reservoirs of orthohantaviruses, since each of them is naturally infected by a particular viral species. However, using the *M*. *musculus* genome as a reference, our transcriptomic analysis by RNA-Seq of infected and non-infected MyglaSwRecB cells revealed significant up regulation of 58 genes by PHV, most of which belong to the IFN pathway, as is the case in human A549 cells infected with PUUV, whereas only a few genes were activated by PUUV infection of bank vole cells. The robustness of our analysis was confirmed by the fact that a similar number of genes was identified when the RNA-Seq reads were mapped to the draft *M*. *glareolus* genome. In addition, the availability of a bank vole genome allowed us to develop RT-qPCR assays to validate the DEG of interest identified in the transcriptomic analysis. In this regard, the newly available bank vole genome represents a promising new resource for future studies aimed at gaining insights into the interaction of PUUV with its natural host.

Altogether, gene regulations described in the present study are consistent with the various outcomes of virus infection. On the one hand, the low expression levels of IFN-induced genes, together with a production of infectious PUUV particles, could parallel persistent infection in the bank vole reservoir. On the other hand, the fact that PHV was able to infect MyglaSWRecB cells, without releasing infectious particles, corroborated the fact that in nature, PHV is not adapted to the bank vole. This was supported by a greater up regulation by PHV, compared to PUUV, of genes from the IFN pathway involved in the antiviral response. Moreover, we have previously reported that in human A549 cells many genes of the IFN pathway were activated by the pathogenic PUUV [30], which is also the case in bank vole cells infected by heterologous non-pathogenic PHV (S1 Table), as shown here. Remarkably, chemokines such as CXCL10 and CXCL11, which are up-regulated in the human A549 cell line infected with PUUV [30] were not detected in the RNA of PUUV-infected bank vole cells. This is consistent with a recently published observation that has been made in a Syrian hamster model of pathogenic and non-pathogenic orthohantavirus infection [83].

In summary, our comparison of different orthohantaviruses in terms of their ability to infect different cell types has shown that they are restricted at different levels of their viral cycle either affecting entry, replication or egress of infectious particles. Our results also show that this relates to the differential interaction of orthohantaviruses with cellular factors. This is evidenced by the distribution of viral-N proteins among cellular sub-compartments and their differential interaction with proteins involved in cellular pathways. Importantly, differences in IFN induction by PUUV and PHV, as well as DEG involved in innate immunity in bank vole and human cells, corroborated the different outcomes of orthohantavirus infections in their different hosts. Our work, combining cellular and molecular approaches, thus highlights the complexity of the interactions of each orthohantavirus with each cell type. By including cell lines derived from the bank vole, the natural reservoir of PUUV, the present study paves the way for further comparative investigations to understand the role of the identified interactions in the differential effects and outcomes of these viruses in their hosts.

The authors are grateful to Nathalie Sauvonnet, Isabella Eckerle, Sandra Essbauer, Karine Badonnel and Dominique Weil, for providing material and support and to Gloria Bua and Corinne Jallet for help with experimental work. The authors thank Elodie Turc and Laure Lemée of the Biomics Platform, IBISA, C2RT, Institut Pasteur, Paris, France, for their great help in sequencing the samples and bioinformatic analysis.

## Supporting information

S1 Fig Infectivity of different cell lines and determination of orthohantavirus infectious titers by immunofluorescence. In the left panels, A1C5 antibody was used to quantify by immunofluorescence the percentage of N+ cells at dpi 3 and dpi 7 in human and bank vole cells infected either with PUUV (A), TULV (B) or PHV (C). For statistical analysis infectivity at dpi7 was compared to the one at dpi 3. Significant differences are added using the same code of p-values as in Fig 1. Non-significant differences are indicated as “ns”. In the right panels, the titration curves of each orthohantavirus are shown. This includes cell lines selected for the present analysis only (Vero E6, HuH7 and MyglaSWRecB), infected with PUUV (A), TULV (B) or PHV (C). Titration curves were obtained by reporting the percentage of infected Vero E6 cells (N+) as a function of the dilution of the supernatants from human HuH7 and bank vole MyglaSWRecB infected cells, compared to their corresponding Vero E6 viral stocks. From these curves, infectious titer in infectious units (IU/mL), were extrapolated and were reported in Fig 2.
(TIF)

S2 Fig Co-localization of viral N protein with markers of the secretory pathway. Individual and merged fluorescence of N labelled in green and, ER (A), ERGIC (B), Golgi (C) and Trans-Golgi network (D) in red, is shown for Vero E6 cells infected with PUUV, TULV or PHV. Nuclei are stained in blue with DAPI. Co-localizing proteins appear in yellow in the merge panel.
(TIF)

S3 Fig Co-localization of viral N protein and viral glycoprotein, Gn. Vero E6 cells infected with PUUV, TULV or PHV were stained for fluorescence. N protein appears in green and Gn in red. Nuclei are stained in blue with DAPI. Co-localizing proteins appear in yellow in the merged panel.
(TIF)

S4 Fig Co-localization of viral N protein with markers of the endocytic pathway. Individual and merged fluorescence of N protein labelled in green and, early endosomes (EE), late endosomes (LE), and recycling of transferrin (tf) in red, in Vero E6 cells infected with PUUV, TULV or PHV, are shown in panel (A), (B) and (C), respectively. Nuclei are stained in blue with DAPI.
(TIF)

S5 Fig Co-localization of viral N protein with cytoskeleton filaments. Individual and merged fluorescence of N labelled in green and actin (A), tubulin (B), and vimentin (C) filaments of the cytoskeleton in red, is shown for Vero E6 cells infected with PUUV, TULV or PHV or non-infected (NI) cells as control. Nuclei are stained in blue with DAPI.
(TIF)

S6 Fig Electron microscopy acquisitions of polarized distribution of viral particles at the cell surface. EM pictures, taken in the same region of Vero E6 cells infected with TULV are shown at different magnification and illustrate the polarized release of full particles and empty tubular structures.
(TIF)

S7 Fig Immunostaining of viral N protein and NKRF or NCBP2. HuH7 cells infected with PUUV, TULV or PHV were proceeded for IF staining of NKRF (A) or NCBP2 (B) appearing in red and the viral nucleocapsid in green. No co-localization of N proteins with these nuclear proteins could be detected as illustrated in the merge panels.
(TIF)

S1 Table RNA-Seq identification of bank vole genes induced by orthohantavirus infection. Up- and down-regulated genes in MyglaSWRecB cells infected by PUUV (Tables A and C) and PHV (Tables B and D) were identified by alignment of the RNA sequences either with *M*. *musculus* (Tables A and B) or *M*. *glareolus* (Tables C and D) genome.
(DOCX)
