# Supplementary figures and images for: Diverse susceptibilities and responses of human and rodent cells to orthohantavirus infection reveal different levels of cellular restriction
Source: PLoS Negl Trop Dis. 2022 Oct 12;16(10):e0010844. doi: 10.1371/journal.pntd.0010844 (PMC9591050; doi:10.1371/journal.pntd.0010844)

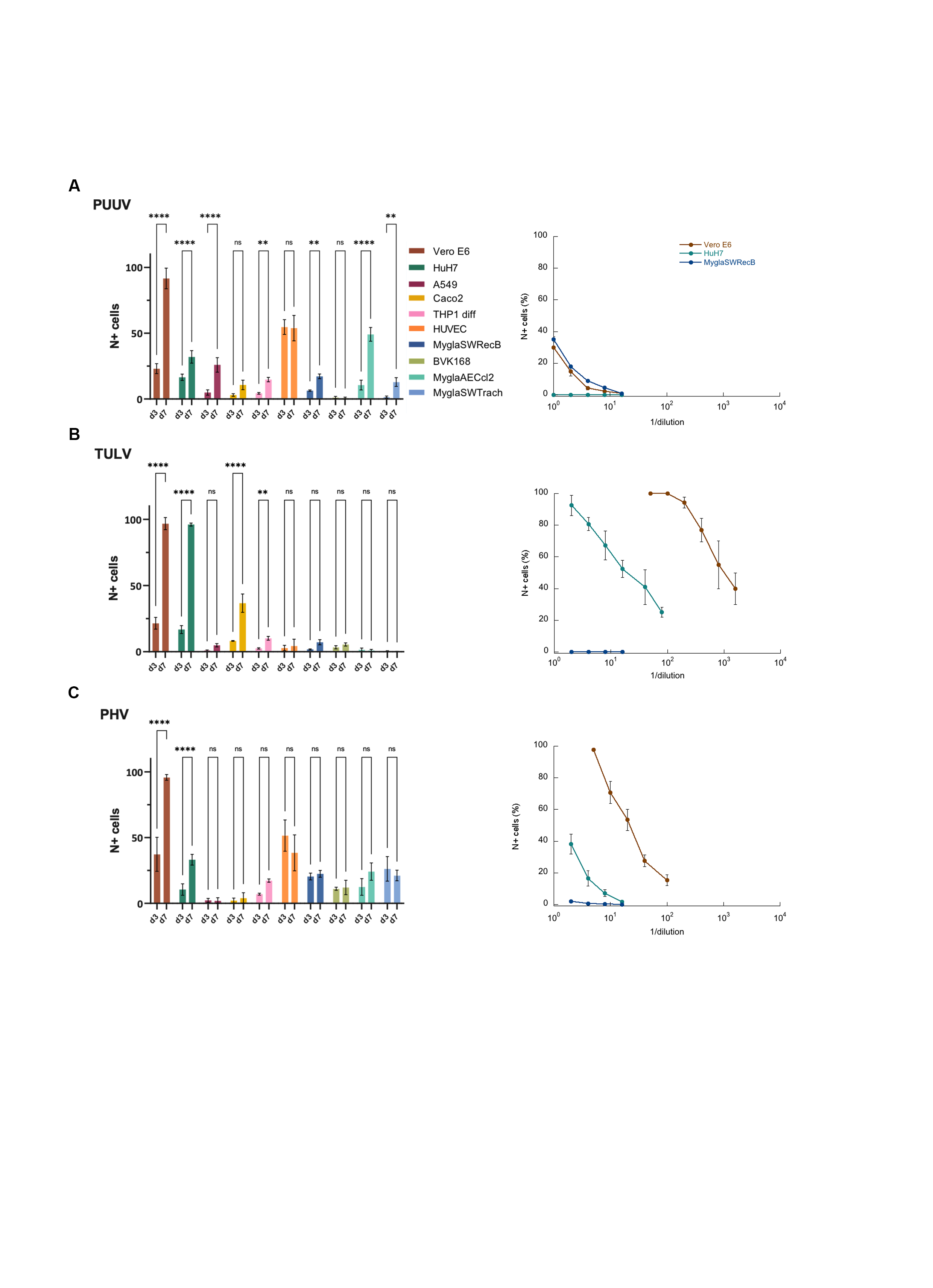

Supplement: S1 Fig — In the left panels, A1C5 antibody was used to quantify by immunofluorescence the percentage of N+ cells at dpi 3 and dpi 7 in human and bank vole cells infected either with PUUV (A), TULV (B) or PHV (C). For statistical analysis infectivity at dpi7 was compared to the one at dpi 3. Significant differences are added using the same code of p-values as in Fig 1. Non-significant differences are indicated as “ns”. In the right panels, the titration curves of each orthohantavirus are shown. This includes cell lines selected for the present analysis only (Vero E6, HuH7 and MyglaSWRecB), infected with PUUV (A), TULV (B) or PHV (C). Titration curves were obtained by reporting the percentage of infected Vero E6 cells (N+) as a function of the dilution of the supernatants from human HuH7 and bank vole MyglaSWRecB infected cells, compared to their corresponding Vero E6 viral stocks. From these curves, infectious titer in infectious units (IU/mL), were extrapolated and were reported in Fig 2. (TIF) [file pntd.0010844.s001.tif]

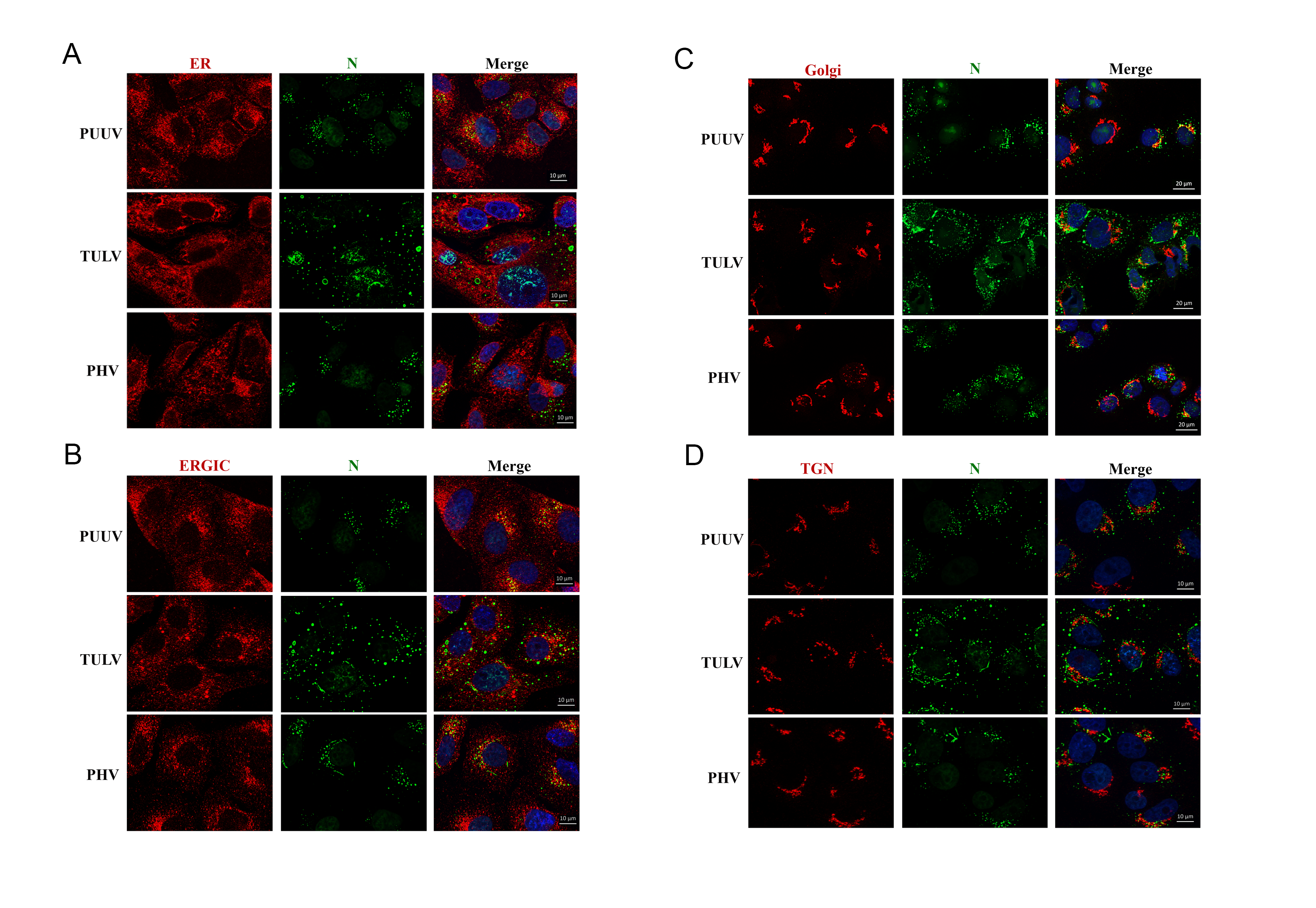

Supplement: S2 Fig — Individual and merged fluorescence of N labelled in green and, ER (A), ERGIC (B), Golgi (C) and Trans-Golgi network (D) in red, is shown for Vero E6 cells infected with PUUV, TULV or PHV. Nuclei are stained in blue with DAPI. Co-localizing proteins appear in yellow in the merge panel. (TIF) [file pntd.0010844.s002.tif]

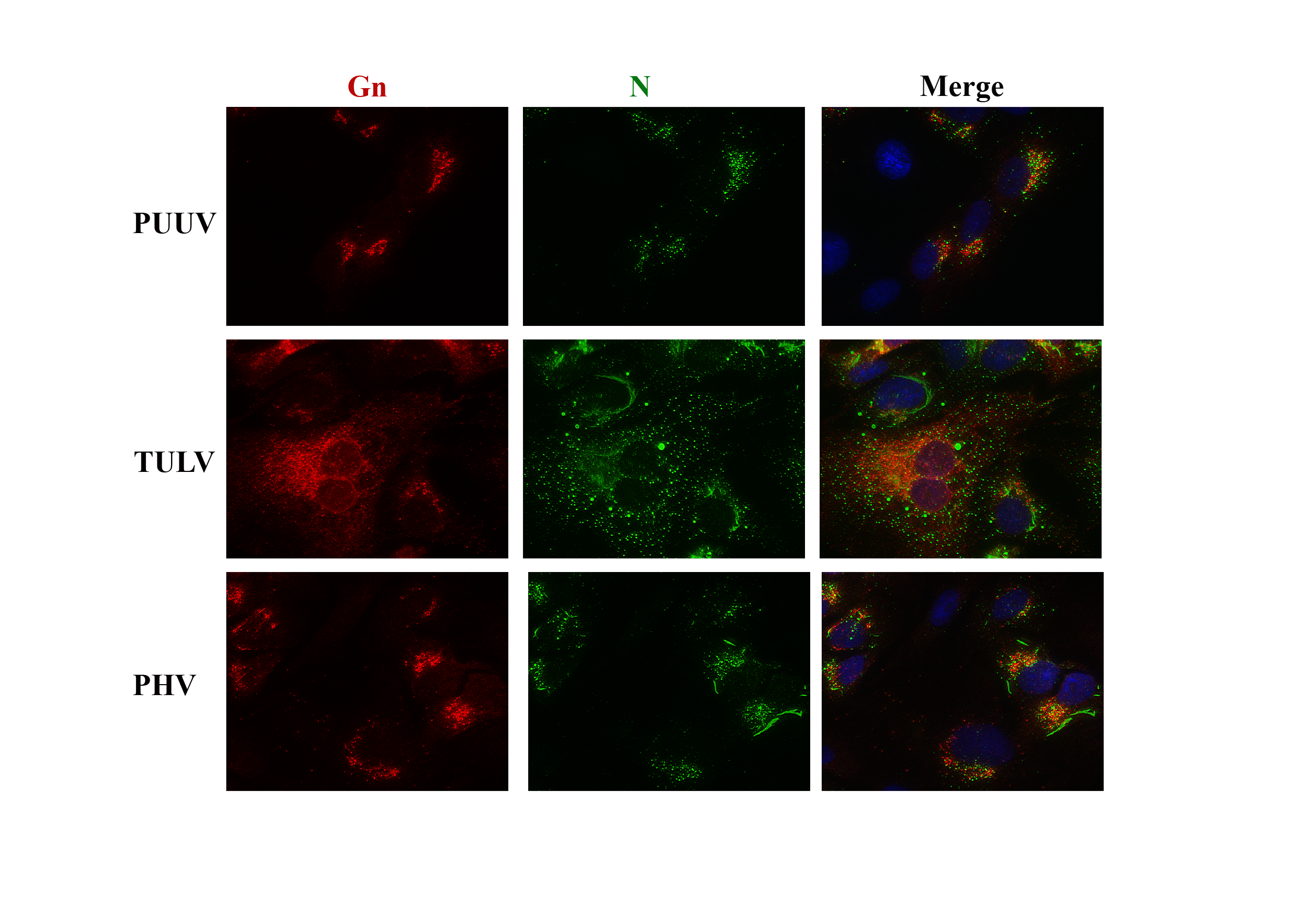

Supplement: S3 Fig — Vero E6 cells infected with PUUV, TULV or PHV were stained for fluorescence. N protein appears in green and Gn in red. Nuclei are stained in blue with DAPI. Co-localizing proteins appear in yellow in the merged panel. (TIF) [file pntd.0010844.s003.tif]

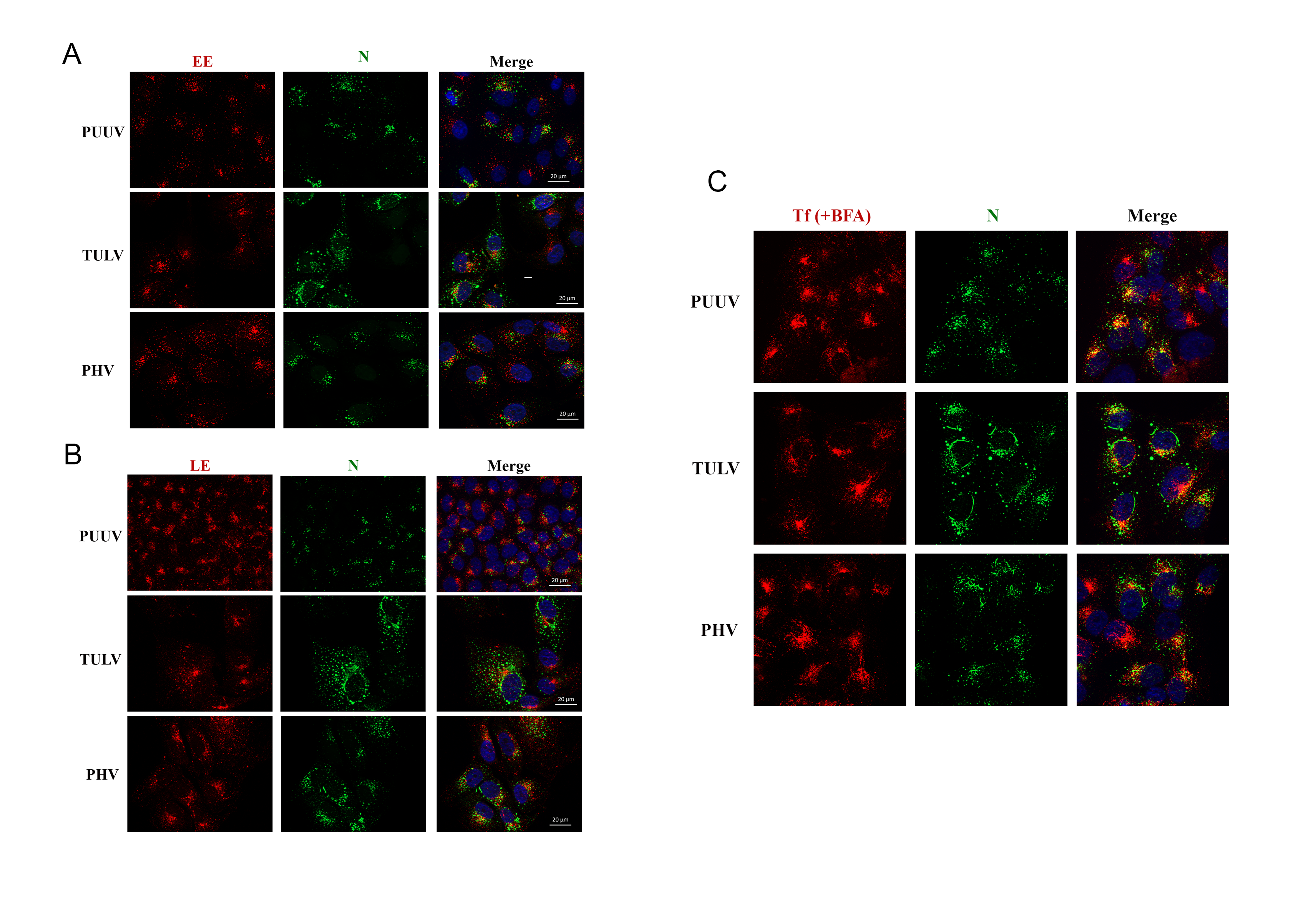

Supplement: S4 Fig — Individual and merged fluorescence of N protein labelled in green and, early endosomes (EE), late endosomes (LE), and recycling of transferrin (tf) in red, in Vero E6 cells infected with PUUV, TULV or PHV, are shown in panel (A), (B) and (C), respectively. Nuclei are stained in blue with DAPI. (TIF) [file pntd.0010844.s004.tif]

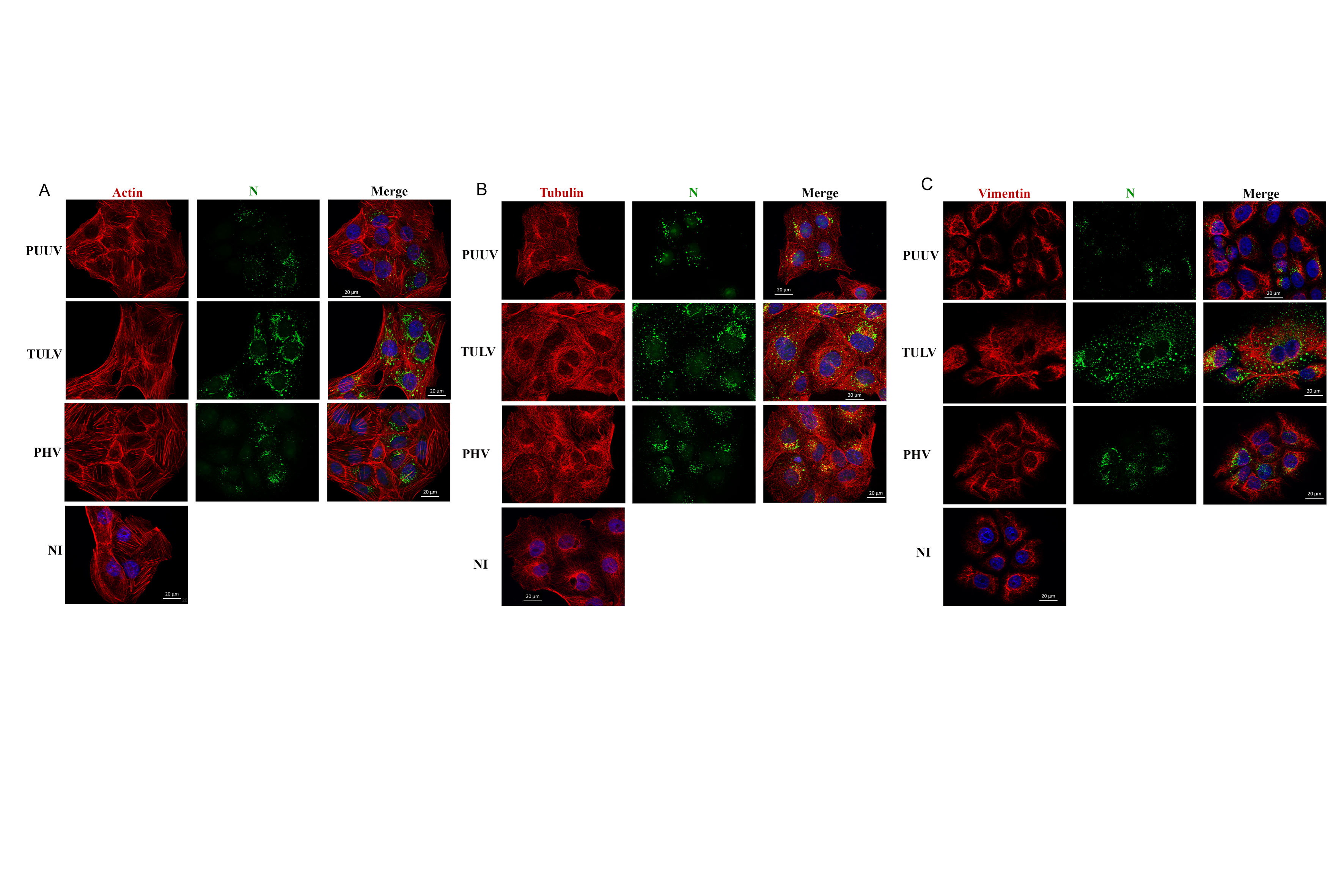

Supplement: S5 Fig — Individual and merged fluorescence of N labelled in green and actin (A), tubulin (B), and vimentin (C) filaments of the cytoskeleton in red, is shown for Vero E6 cells infected with PUUV, TULV or PHV or non-infected (NI) cells as control. Nuclei are stained in blue with DAPI. (TIF) [file pntd.0010844.s005.tif]

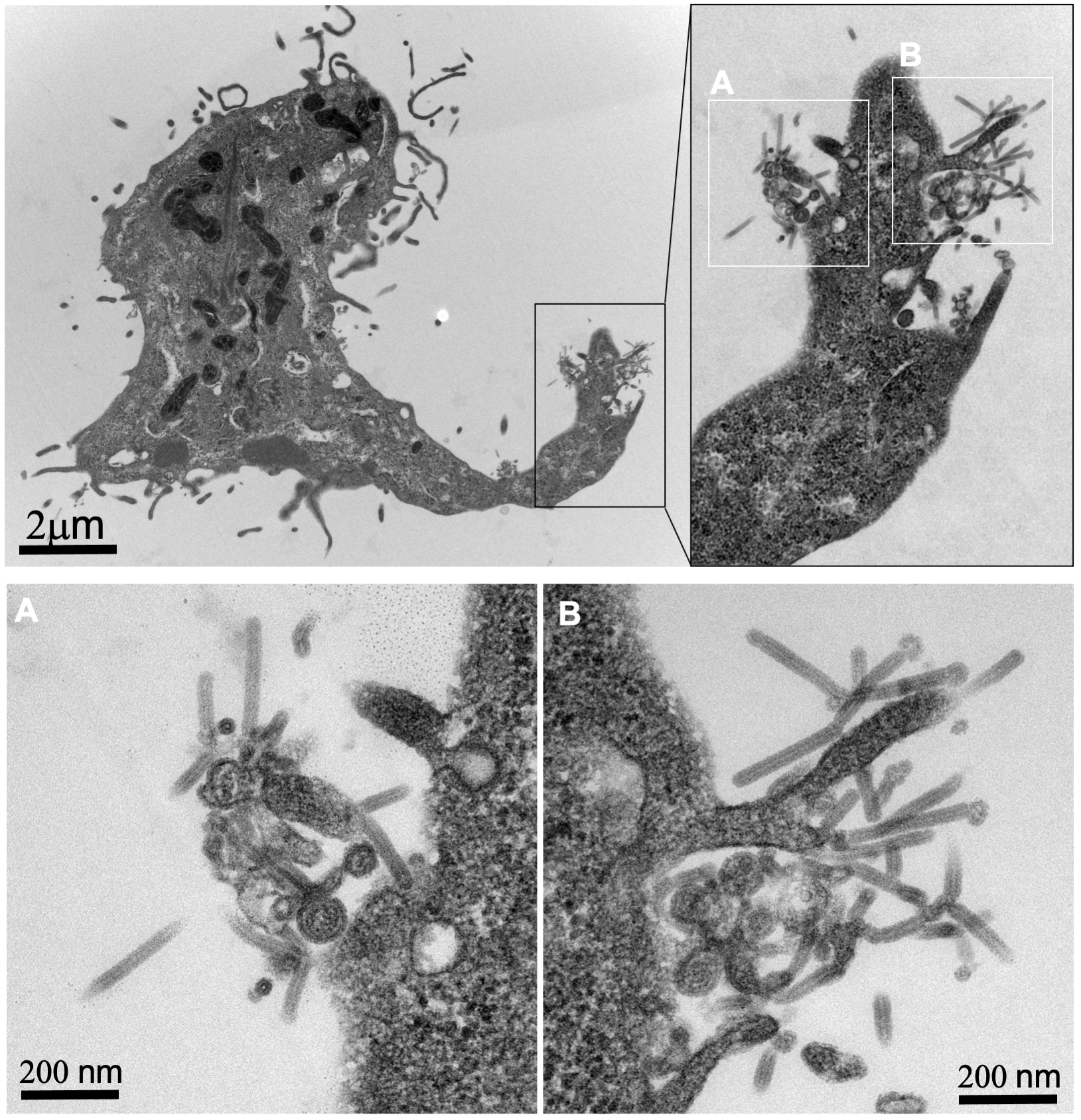

Supplement: S6 Fig — EM pictures, taken in the same region of Vero E6 cells infected with TULV are shown at different magnification and illustrate the polarized release of full particles and empty tubular structures. (TIF) [file pntd.0010844.s006.tif]

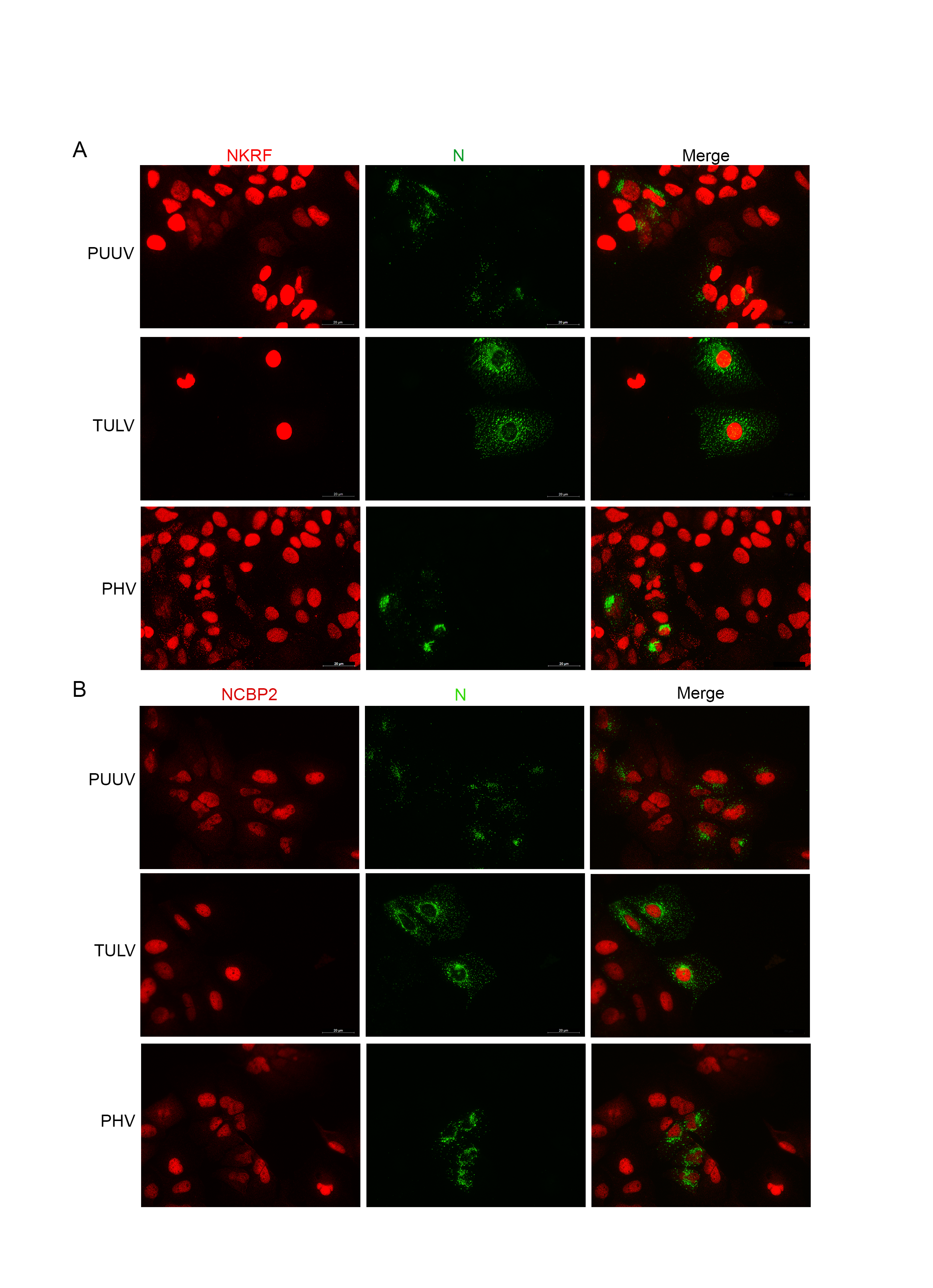

Supplement: S7 Fig — HuH7 cells infected with PUUV, TULV or PHV were proceeded for IF staining of NKRF (A) or NCBP2 (B) appearing in red and the viral nucleocapsid in green. No co-localization of N proteins with these nuclear proteins could be detected as illustrated in the merge panels. (TIF) [file pntd.0010844.s007.tif]
