# Supplementary material for: Diverse susceptibilities and responses of human and rodent cells to orthohantavirus infection reveal different levels of cellular restriction
Source: PLoS Negl Trop Dis. 2022 Oct 12;16(10):e0010844. doi: 10.1371/journal.pntd.0010844 (PMC9591050; doi:10.1371/journal.pntd.0010844)
Supplement: S1 Table — Up- and down-regulated genes in MyglaSWRecB cells infected by PUUV (Tables A and C) and PHV (Tables B and D) were identified by alignment of the RNA sequences either with M. musculus (Tables A and B) or M. glareolus (Tables C and D) genome. (DOCX) [file pntd.0010844.s008.docx]

**S1 Table A: Upregulated (N=8) and downregulated (N=1) genes in PUUV-infected MyglaSWRecB cells identified by alignment of RNA-Seq reads with the reference genome of *Mus musculus***

| **Gene Name^1^** | **fold change^2^** | **Anova. P<0.05** | **Function** |
| --- | --- | --- | --- |
| Gipr | 7.194 | 1.864e-8 | GPCR signaling |
| Ifit1 | 2.746 | 7.230e-6 | Inhibition of viral replication and translation |
| Stab1 | 2.525 | 5.754e-5 | Stabilin LDL receptor |
| Vav1 | 2.501 | 1.664e-9 | Oncogene |
| Gbp7 | 2.32 | 5.927e-5 | GTPase; cellular response to IFNγ |
| Mx2 | 2.13 | 9.41e-17 | IFI, antiviral response |
| Inpp5d | 2.108 | 2.433e-5 | phosphatase; neutrophil migration; apoptotic process |
| Hcls1 | 2.063 | 4.907e-5 | receptor signaling (Mitochondria) |
|  |  |  |  |
| Mrpl1 | 0.3273 | 1.781e-5 | Mitochondrial 39S ribosomal protein; traduction |

^1^ Genes found upregulated both by PUUV and PHV infection of bank vole cells appear in violet and in green when found also in human (ref 30). ^2^ Genes upregulated with a FC >2 and downregulated genes with a FC< 0.5 relative to non-infected cells are shown.

**S1 Table B: Upregulated (N=58) and downregulated (N=9) genes in PHV infected MyglaSWRecB cells identified by alignment of RNA-Seq reads with the reference genome of *M musculus***

| **Gene Name** | **fold change** | **Anova. P<0.05** | **Function** |
| --- | --- | --- | --- |
| Gm4951 | 57.74 | 2.007e-14 | ER membrane GTPase IFNγ induced (cf Irgm1) |
| Iigp1* | 37.03 | 1.447e-15 | IFI GTPase |
| Ifit1* | 24.33 | 1.197e-54 | Inhibition of viral replication and translation |
| Rsad2* | 23.3 | 2.197e-26 | IFI antiviral state: inhibitor of viral replication |
| Ifit1bl1 | 18.01 | 1.257e-19 | IFI cellular response to viruses |
| Oasl1* | 14.39 | 2.643e-30 | Antiviral activity independent of RNase L |
| Gbp7 | 13.96 | 3.225e-43 | IFI GTPase; cellular response to IFNγ |
| Ifit2* | 13.61 | 2.518e-70 | IFI-antiviral defense; apoptosis; Inhibition of viral transcription |
| Mx2 | 13.22 | 1.986e-187 | IFI, antiviral response |
| Gbp11 | 12.27 | 2.583e-38 | GTPase; cellular response to IFNγ |
| Mx1 | 10.18 | 1.444e-10 | IFI antiviral response |
| Oas1g | 9.835 | 4.252e-8 | Defense response to virus |
| Uba7* | 9.948 | 1.119e-17 | ISG15 Ubiquitin modification: negative regulation IFN-I |
| Oas3* | 8.137 | 3.347e+4 | IFI dsRNA antiviral activity |
| Gbp8 | 7.967 | 8.171e-7 | GTPase; cellular response to IFNγ |
| Irf7 | 6.909 | 7.053e-19 | IFN regulatory transcription factor |
| Lgals9 | 6.77 | 1.008e-24 | Galectin=lectin modulating cell interaction |
| Cmpk2 | 6.551 | 2.733e-8 | Mitochondrial nucleotide biosynthetic process |
| Oas1a* | 6.329 | 1.092e-4 | IFI-defense response to virus |
| Usp18 | 5.734 | 2.127e-5 | Negative regulator of IFN-I signaling pathway; ISGylaton |
| Ddx60* | 5.441 | 5.198e-18 | RNA helicase: regulates IFN induced by RIG & MDA5 |
| Dhx58 | 5.052 | 1.085e-24 | Regulates RIG1 mediated antiviral signaling |
| Irgm1* | 4.909 | 2.518e-5 | GTPase: regulates IFNγ, proinflammatory cytokines, autophagy |
| Parp14* | 4.788 | 2.557e-36 | ADP ribosyl transfer on glutamate, IIR |
| Pfp1 | 4.441 | 5.379e-5 | Macrophage gene 1, transmembrane protein of cytoplasmic vesicles; defense response |
| Ddx58* | 4.336 | 8.144e-14 | RIG-I, RNA helicase promoting IFN signaling |
| Ifih1* | 4.33 | 2.16e-23 | MDA5, RLR^2^ signaling |
| Slfn4 | 3.69 | 2.606e-26 | Upregulated by IFN-I signaling, regulate immune cells |
| Ube2l6* | 3.499 | 1.427e-12 | Ubiquitinylation of proteins such as ISB15, p53 |
| Ly6e* | 3.48 | 1.674e-19 | GPI mb protein: restricts viral entry/regulate TCR signal |
| Irf9* | 3.369 | 8.341e-29 | Transcription factor: role in antiviral immunity |
| Stat1* | 3.257 | 1.385e-21 | IFN -I signaling: defense response to virus |
| H2-Q1 | 3.247 | 2.511e-6 | Mouse MHC-I, antigen presentation and signaling |
| SAMD9L* | 3.127 | 3.51e-35 | Early endosome; response to viral infection; endosome fusion |
| Epsti1 | 3.188 | 3.35e-10 | Epithelial stromal interaction |
| Ifitm3* | 3.115 | 1.061e-9 | IFI antiviral mb protein: inhibits viral fusion |
| Parp12 | 3.024 | 1.751e-37 | Nuclear transferase mediating ADP-ribosylation |
| Trim14 | 2.855 | 3.118e-4 | Ub ligase; IIR; autophagy: roles in viral entry and transcription |
| C1s2 | 2.837 | 4.861e-6 | Complement component activation; endopeptidase |
| Parp9 | 2.788 | 3.808e-6 | Role in IIR including IFN-mediated antiviral defenses |
| Helz2* | 2.744 | 5.838e-43 | Helicase with Zn finger domain 2/peroxisome activation |
| Stat2* | 2.713 | 1.761e-20 | IFN -I signaling: defense response to virus |
| Fcgr1 | 2.683 | 5.71e-4 | Fc receptor; antigen presentation; phagocytosis |
| Rnf213* | 2.397 | 1.91e-51 | E3 ubiquitin ligase involved in angiogenesis |
| Ttc39c | 2.39 | 3.866^e^-7 | Tetratricopeptide repeat domain 39C; Unknown function |
| Unc93b1 | 2.263 | 3.34e-11 | Regulator of TLR signaling |
| Cds1 | 2.254 | 9.123e-5 | Role in growth of lipid droplets: PI and PG synthesis |
| Crmp1 | 2.234 | 3.17e-4 | Cytoskeleton remodeling; regulation of actin filament binding |
| Adar* | 2.214 | 2.22e-68 | protection against virus induced cytopathic effects |
| Tap1 | 2.196 | 1.645e-19 | ABC-transporter: Ag presentation via MHC-I |
| Trim21 | 2.183 | 0.0002213 | Ubiquitinylation; cell cycle; inhibits viral release; induce autophagy |
| H2-M3 | 2.181 | 4.813e-22 | Mouse MHC-I, antigen presentation and signaling |
| Dtx3l | 2.154 | 7.065e-6 | Ubiquitin ligase; defense response to virus |
| Psmb8 | 2.111 | 2.751e-14 | Proteasome subunit: endopeptidase |
| Vav1 | 2.102 | 1.589e-6 | Guanine exchange factor: role in cytoskeleton rearrangment |
| Mpeg1* | 2.089 | 5.923e-8 | Macrophage expressed (perforin), IIR |
| Ifi35 | 2.08 | 4.644e-10 | Signaling in innate immune response with IFNα |
| Acta2* | 2.015 | 2.624e-7 | response to virus; ERK regulation |
|  |  |  |  |
| Timm23 | 0.0483 | 2.54e-4 | Mitochondrial import |
| Rps27a | 0.2943 | 1.52e-4 | Ubiquitin; role in ERAD, endocytosis; NFκB activation, signaling |
| Cd82 | 0.3083 | 4.90e-4 | Membrane antigen associates to CD4/CD8 |
| Cox20 | 0.3607 | 3.75e-4 | Assembly of mitochondrial cytochrome c assembly |
| Etfrf1 | 0.4207 | 3.91e-4 | Mitochondrial respiratory transport chain |
| Rpl26 | 0.4214 | 3.88e-5 | 60S ribosomal protein, regulation of intrinsic apoptosis |
| Tctex1d2 | 0.4323 | 3.74e-4 | Dynlt2b in mouse; acts on dynein complex; microtubule transport |
| Map7 | 0.4817 | 4.00e-5 | Microtubule stabilizing protein |
| Msmp | 0.4992 | 4.44e-6 | CCR2 ligand: chemotactic activity |

^1^ Genes found upregulated both by PUUV and PHV infection of bank vole cells appear in violet and in green when found also in human (ref 30). In blue are the genes found upregulated in bank vole cells by PHV and in human cells by PUUV. * indicates the genes identified by alignment with both reference (*Mus musculus* and *Myodes glareolus*). ^2^ Genes upregulated with a FC >2 and downregulated with a FC< 0.5 compared to non-infected cells are shown.

**S1 Table C: Upregulated genes (N=6) in PUUV-infected MyglaSWRecB cells identified by alignment of RNA-Seq reads with the reference genome of *Myodes glareolus***

| **Gene Name^1^** | **fold change^2^** | **Anova. P<0.05** | **Function** |
| --- | --- | --- | --- |
| Ednrb | 2.468 | 1.601e-5 | Angiotensin receptor GPCR |
| Unknown | 2.453 | 5.54e-12 | Unknown |
| Iigp1 | 2.288 | 9.22e-12 | IFI GTPase |
| Msr1 | 2.268 | 6.03e-10 | Endocytosis & phagocytosis: LDL transport regulation |
| Mpeg1 ﻿ | 2.187 | 1.070e-8 | Macrophage expressed (perforin) innate immune response |
| Alox5ap | 2.044 | 1.177e-5 | protein trimerization; positive regulation of inflammatory response |

^1^ “Unknown” stands for genes not present in the reference annotation. Genes found upregulated both by PUUV and PHV infection of bank vole cells appear in violet . ^2^ Genes upregulated with a FC >2 and downregulated genes with a FC< 0.5 relative to non infected cells are shown.

**S1 Table D : Upregulated genes (N=57) and downregulated (N=5) in PHV-infected MyglaSWRecB cells identified by alignment of RNA-Seq reads with the reference genome of Myodes glareolus**

| **Gene Name^1^** | **fold change^2^** | **Anova. P<0.05** | **Function** |
| --- | --- | --- | --- |
| Iigp1 * | 28.9 | 1.1e-198 | Interferon-inducible GTPase 1 |
| Rsad2* | 19.2 | 1.1e-156 | IFI antiviral state: inhibitor of viral replication |
| Unknown | 18.67 | 1.4e-126 | Unknown |
| IFIT2* | 13.63 | 1.58e-28 | IFI-antiviral defense; apoptosis; defense response to virus |
| Ifi203 | 13.58 | 3.22e-68 | IFNβ-activation p 203 innate immune response |
| Oas3* | 12.88 | 2.250e-5 | IFI dsRNA antiviral activity |
| Herc6 | 12.06 | 6.54e-20 | ISG15 ubiquitin conjugation: regulator of innate antiviral immunity |
| Unknown | 11.64 | 7.31e-36 | Unknown |
| Cfb | 11.11 | 5.598e-5 | Complement alternate system; cell proliferation |
| Oasl* | 9.92 | 3.07e-87 | Antiviral activity independent of RNase L |
| Cd274 | 9.545 | 1.455e-4 | Ligand for the inhibitory receptor PDCD1: adaptative tolerance (mb, EE, recycling Endosome) |
| Uba7* | 9.469 | 7.67e-23 | ISG15 Ubiquitin modification: negative regulation IFN-I |
| Ifit1* | 8.104 | 1.591e-9 | Inhibition of viral replication and translation |
| Irgm1* | 7.469 | 3.21e-52 | GTPase: regulates IFNγ, proinflammatory cytokines, autophagy |
| Parp14* | 6.374 | 5.696e-7 | ADP ribosyl transfer on glutamate, innate immune response |
| Ddx60* | 4.976 | 4.10e-15 | RNA helicase: promoting IFN signaling induced by RIG & MDA5 |
| Sp100 | 4.886 | 3.27e-20 | Human nuclear antigen Sp100 |
| Perm1 | 4.74 | 1.974e-4 | Regulation of transcription: glucose and lipid metabolism |
| Ddx58* | 4.538 | 1.13e-40 | RIG-I, RNA helicase promoting IFN signaling |
| Unknown | 4.279 | 2.766e-5 | Unknown |
| SAMD9* | 4.226 | 3.81e-33 | Inflammatory response; target of TNFα signaling, endosome fusion |
| Oas1a* | 4.189 | 1.64e-33 | IFI-defense response to virus |
| Nlrc5 | 4.06 | 2.338e-6 | Regulator NFkB & IFN: control antiviral defense |
| Ifih1* | 3.946 | 4.83e-22 | RIG-I, RNA helicase promoting IFN signaling |
| Unknown | 3.821 | 3.64e-17 | Unknown |
| Ube2l6* | 3.394 | 2.52e-15 | Ubiquitinylation of proteins such as ISB15, p53 |
| Unc5b | 3.28 | 5.427e-4 | Netrin receptor: angiogenesis and regulation of apoptosis |
| Tnfsf10 | 3.276 | 2.05e-25 | Cytokine involved in apoptosis |
| Unknown | 3.247 | 2.61e-38 | Unknown |
| Stat1* | 3.146 | 3.04e-48 | IFN -I signaling: defense response to virus |
| Ly6e* | 3.124 | 1.17e-67 | GPI mb prot/restric viral entry/regulate TCR signal |
| Trim30a | 3.113 | 8.54e-13 | Regulates IL2Ra: (-) regulation IL6, TLR, TNF; autophagy (+) regulation |
| PPP1R8 | 3.039 | 5.135e-4 | Nuclear inhibitor of phosphatase 1; mRNA processing |
| Helz2* | 2.987 | 2.37e-28 | Helicase with Zn finger domain 2/peroxisome activation |
| Ifitm3* | 2.797 | 2.99e-14 | IFN-induced antiviral mb protein: inhibits viral fusion |
| Stat2* | 2.674 | 5.24e-29 | IFN -I signaling: defense response to virus |
| Acta2* | 2.642 | 3.424e-4 | Actin:glomerular development; response to virus; ERK regulation |
| Rnf213* | 2.596 | 2.43e-17 | E3 ubiquitin ligase involved in angiogenesis |
| Unknown | 2.584 | 7.69e-49 | Unknown |
| Mpeg1* | 2.555 | 2.363e-7 | Macrophage expressed (perforin) innate immune response |
| Unknown | 2.548 | 1.47e-12 | Unknown |
| IRF9* | 2.51 | 1.05e-12 | Interferon regulatory factor 9 |
| SP140 | 2.454 | 3.382e-9 | Nuclear protein / involved in viral defense |
| Xaf1 | 2.432 | 4.18e-25 | IFNβ insduced: Negative regulator of apoptose inhibitor |
| Msr1 | 2.419 | 2.68e-11 | Endocytosis & phagocytosis: LDL transport regulation |
| Cadps | 2.385 | 5.41e-23 | serine proteinase (ref Syrian Hamster) |
| Gmpr1 | 2.38 | 4.22e-24 | conversion of nucleobase, nucleoside and nucleotide |
| C4 | 2.36 | 9.780e-6 | Complement; inflammatory response; apoptotic cell clearance regulation |
| Trim12a | 2.353 | 2.81e-12 | Ubiquitin activity; autophagy |
| Unknown | 2.353 | 1.914e-4 | Unknown |
| H2-Q10 | 2.29 | 7.43e-11 | MHC class 1; peptide presentation (increased in hantavirus infected cells |
| Unknown | 2.231 | 3.65e-12 | Unknown |
| Unknown | 2.23 | 1.24e-10 | Unknown |
| Unknown | 2.188 | 4.168e-8 | Unknown |
| Adar* | 2.181 | 1.03e-31 | ds RNA deamination: protection against virus induced cytopathic effects |
| Lgals3bp | 2.131 | 4.41e-24 | Promotes integrin cell adhesion; stimulates viral defense |
| RNAse4 | 2.115 | 4.18e-11 | Endonuclease: response to starvation |
|  |  |  |  |
| Ces1d | 0.3124 | 8.47e-5 | Carboxylesterase: fatty acid synthesis |
| Unknown | 0.3689 | 5.63e-5 | Unknown |
| Micos13 | 0.4104 | 2.71e-4 | Component of mitochondrial inner membrane complex |
| Unknown | 0.4364 | 5.15e-4 | Unknown |
| Inmt | 0.4729 | 4.97e-6 | N-methyltransferase: response to toxic substance |

^1^ ^1^ “Unknown” stands for genes not present in the reference annotation. Genes found upregulated both by PUUV and PHV infection of bank vole cells appear in violet and in green when found also in human (ref 30). In blue are the genes found upregulated in bank vole cells by PHV and in human cells by PUUV. * indicates the genes identified by alignment with both reference (*Mus musculus* and *Myodes glareolus*). ^2^ Genes upregulated with a FC >2 and downregulated with a FC< 0.5 compared to non-infected cells are shown.
